# A Mindfulness-Based Intervention for Student Depression, Anxiety, and Stress: Randomized Controlled Trial

**DOI:** 10.2196/23491

**Published:** 2021-01-11

**Authors:** Paul Ritvo, Farah Ahmad, Christo El Morr, Meysam Pirbaglou, Rahim Moineddin, MVC Team

**Affiliations:** 1 School of Kinesiology and Health Science Faculty of Health York University Toronto, ON Canada; 2 School of Health Policy and Management York University Toronto, ON Canada; 3 Dalla Lana School of Public Health University of Toronto Toronto, ON Canada; 4 York University Toronto, ON Canada

**Keywords:** online intervention, randomized controlled trial, university student, depression, anxiety, stress, mental health, efficacy, intervention

## Abstract

**Background:**

University students are experiencing higher levels of distress and mental health disorders than before. In addressing mental health needs, web-based interventions have shown increasing promise in overcoming geographic distances and high student-to-counselor ratios, leading to the potential for wider implementation. The Mindfulness Virtual Community (MVC) program, a web-based program, guided by mindfulness and cognitive behavioral therapy principles, is among efforts aimed at effectively and efficiently reducing symptoms of depression, anxiety, and perceived stress in students.

**Objective:**

This study’s aim was to evaluate the efficacy of an 8-week MVC program in reducing depression, anxiety, and perceived stress (primary outcomes), and improving mindfulness (secondary outcome) in undergraduate students at a large Canadian university. Guided by two prior randomized controlled trials (RCTs) that each demonstrated efficacy when conducted during regular university operations, this study coincided with a university-wide labor strike. Nonetheless, the students’ response to an online mental health program on a disrupted campus can provide useful information for anticipating the impact of other disruptions, including those related to the COVID-19 pandemic as well as future disruptions.

**Methods:**

In this parallel-arm RCT, 154 students were randomly allocated to an 8-week MVC intervention (n=76) or a wait-list control (WLC) condition (n=78). The MVC intervention included the following: (1) educational and mindfulness video modules, (2) anonymous peer-to-peer discussions, and (3) anonymous, group-based, professionally guided, 20-minute videoconferences. Study outcomes were evaluated at baseline and at 8-week follow-up using the following: Patient Health Questionnaire-9 (PHQ-9), the Beck Anxiety Inventory (BAI), the Perceived Stress Scale (PSS), and the Five Facets Mindfulness Questionnaire Short Form (FFMQ-SF). Generalized estimation equations with an AR (1) covariance structure were used to evaluate the impact of the intervention, with outcome evaluations performed on both an intention-to-treat (ITT) and per-protocol (PP) basis.

**Results:**

Participants (n=154) included 35 males and 117 females with a mean age of 23.1 years. There were no statistically significant differences at baseline between the MVC and WLC groups on demographics and psychological characteristics, indicating similar demographic and psychological characteristics across the two groups. Results under both ITT and PP approaches indicated that there were no statistically significant between-group differences in PHQ-9 (ITT: β=–0.44, *P*=.64; PP: β=–0.62, *P*=.053), BAI (ITT: β=–2.06, *P*=.31; PP: β=–2.32, *P*=.27), and FFMQ-SF (ITT: β=1.33, *P*=.43; PP: β=1.44, *P*=.41) compared to WLC. There was a significant difference for the PSS (ITT: β=–2.31, *P*=.03; PP: β=–2.38, *P*=.03).

**Conclusions:**

During a university labor strike, the MVC program led to statistically significant reductions in PSS compared to the WLC group, but there were no other significant between-group differences. Comparisons with previous cycles of intervention testing, undertaken during nondisrupted university operations, when efficacy was demonstrated, are discussed.

**Trial Registration:**

ISRCTN Registry ISRCTN92827275; https://www.isrctn.com/ISRCTN92827275

## Introduction

The impact of COVID-19 and other disruptions on education, generally, and secondary education, specifically, can have major student mental health effects. COVID-19–related shutdowns of face-to-face high school education have been associated with significant student dropout rates in multiple cities in the United States, as high proportions of students, engaged face-to-face, failed to connect online (13%) [1]. With the escalating infection rates of both spring and fall 2020, many university courses that were initiated in face-to-face formats were completed online [2,3], with considerable uncertainties about the fall 2020 semester and the impact of COVID-19 outbreaks on campus life generally.

However, the response of students to other campus crises can provide useful data in anticipating the effects of other disruptions. In 2018, during a 4-year project assessing the effects of mindfulness-based cognitive behavioral therapy (M-CBT), York University was impacted by a faculty and teaching assistant strike. Most students respected picket lines and did not attend classes until the strike resolved, which occurred after the end of the usual calendar-defined semester. Despite the strike, study recruitment and intervention testing proceeded. Here, we report the results of that randomized controlled trial (RCT).

The RCT undertaken was identical to two prior studies [4,5] on the same campus site under noncrisis conditions. As the studies were undertaken months apart, there were minimal environmental differences, other than seasonal change. In the initial study [4], 113 students (mean age 24.8 years) participated in an 8-week online M-CBT program and significant between-group benefits were found on measures of depression (Patient Health Questionnaire-9 [PHQ-9]), anxiety (Beck Anxiety Inventory [BAI]), quality of life (Quality of Life Scale [QOLS]), and mindfulness (Five Facet Mindfulness Questionnaire - Short Form [FFMQ-SF]), favoring the intervention group (MVC) compared to wait-list controls (WLC). In the follow-up study [5], 159 students (mean age 22.5 years) participated in the same 8-week online M-CBT program and significant between-group differences were again found on identical measures of depression (PHQ-9), anxiety (BAI), mindfulness (FFMQ-SF), and quality of life in intervention participants, compared to wait-list controls. These two demonstrations of intervention efficacy prepared us to investigate how the campus crisis may have affected student responses.

All three studies were motivated by the rising prevalence of mental health disorders, including depression and anxiety, among college students worldwide, prior to the COVID-19 pandemic [6]. In the United States, for example, analyses of college data show that mental health disorders are among the top 5 diagnostic categories seen at college health services and are responsible for the highest number of visits (4.93) per student [7]. Multiple US studies have suggested there is an increasing prevalence of mental health disorders, especially depression and anxiety, among undergraduate college and university students [7-14].

Additionally, of concern for online researchers, there are possible links between decreased youth mental health and increased online activity (ie, “screen time”) [15-17]. Strong arguments and reliable data support the perspective that online activities, particularly social media engagement, have unhealthy impacts for some youth populations [15-17]. Disruptions of regular face-to-face classes at universities and high schools may have elevated levels of unhealthy “screen time” for select populations. Accordingly, it is worthwhile to ascertain what changes might have occurred when university students experiencing a disruption that likely increased time spent online used an online mental health intervention.

A student survey of 32 Canadian postsecondary institutions indicated a high prevalence of high anxiety (56.5%), hopelessness (54%), seriously depressed mood (37.5%), and overwhelming anger (42%) [18]. The mental health problems seen among North American students are also apparent worldwide, as the World Health Organization (2018) reported increasing mental disorders in college and university students [6].

Despite student distress, the face-to-face counseling offered in colleges and universities has not kept pace with demand. For example, from 2007 to 2012, full-time enrollment in the Ontario (Canada) college system increased by 26% while the number of counselors employed in the college system increased by only 4.6% [19]. This discrepancy has resulted in observations of underserved students and overwhelmed counselors amid the increasing distress among students.

Mindfulness-based interventions have been demonstrated to positively impact psychological and physical health [20-22], with several meta-analyses demonstrating impacts across clinical and nonclinical populations [22-27]. However, with large numbers of students (50,000-60,000 on some campuses), there may not be sufficient numbers of trained personnel to convey helpful mindfulness-based practices directly.

Accordingly, we developed a web-delivered M-CBT program (aimed at creating a Mindfulness Virtual Community, or MVC) to reduce depression, anxiety, and stress in university students and, as mentioned above, previously reported on two RCTs targeting Canadian university students that indicated efficacy [4,5].

## Methods

### Trial Design and Ethical Approval

This study was a two-arm parallel-design RCT comparing the web-based Mindfulness Virtual Community program to a wait-list control group. The Human Participant Research Committee at York University provided research ethics approval for the RCT (Certificate number: e2016 - 345).

### Participants and Recruitment

Eligibility criteria were applied to recruit actively enrolled undergraduates aged ≥18 years, with English-language fluency and self-reported confidence in completing the study. Students were excluded if they reported substance abuse or episodes of psychosis during the month prior to the trial.

The study was advertised using study posters, class announcements, and email invitations via listservs of student associations in the Faculties of Health and Liberal Arts. Interested students contacted the research staff via email or phone and were screened for student registration, substance abuse, and indications of psychoses. If abuse or psychotic behaviors “interfered in routine life within the last month,” students were excluded and provided with a list of accessible mental health resources. In addition, a registered clinical psychologist could be contacted directly if there was a perceived need for interim mental health counseling.

Eligible and willing students received detailed in-person information about the study and provided informed written consent. Participants had the option to receive an honorarium of Can $50 (US $39) or 2% in course grade (for professors who gave permission for this option) or three credits (equivalent to 2% course grade) in the Undergraduate Research Participation Pool (URPP) of the Department of Psychology. Each participant also received a resource list that included information about health and social services on campus and in the community (eg, the 24/7 “Good to Talk” helpline for postsecondary students in Ontario). Our protocol included a safety mechanism whereby participants were asked verbally and on the consent form to contact the research staff if they felt distress during the trial period so that “limited counselling with a clinical psychologist could be arranged, if needed.” No such requests arose during the reported study period.

A sample of 480 students (160 students per group) was recruited over 3 semesters (Fall 2017, Winter 2018, and Fall 2018). The 3 samples were not combined due to the campus environment differences related to the 3-month strike in Winter 2018. Here, we report on a sample of n=154 students in a two-arm RCT.

### Randomization

Participating students were randomized to the MVC intervention or the wait-list (control) using 1:1 block randomization. The randomized allocation sequence was computer-generated by an off-site research team member and allocations were concealed in sequentially numbered opaque envelopes [28]. The envelopes were only opened after written consent was obtained, ensuring participants and staff were blind prior to the allocation. Each participant in the MVC group received a unique ID and a temporary password; participants changed passwords after their first login, while IDs remained the same to reduce the potential of multiple accounts or identities. Participants in all groups completed online questionnaires at baseline (T1) and 8 weeks (T2).

### Intervention

**Figure 1:**
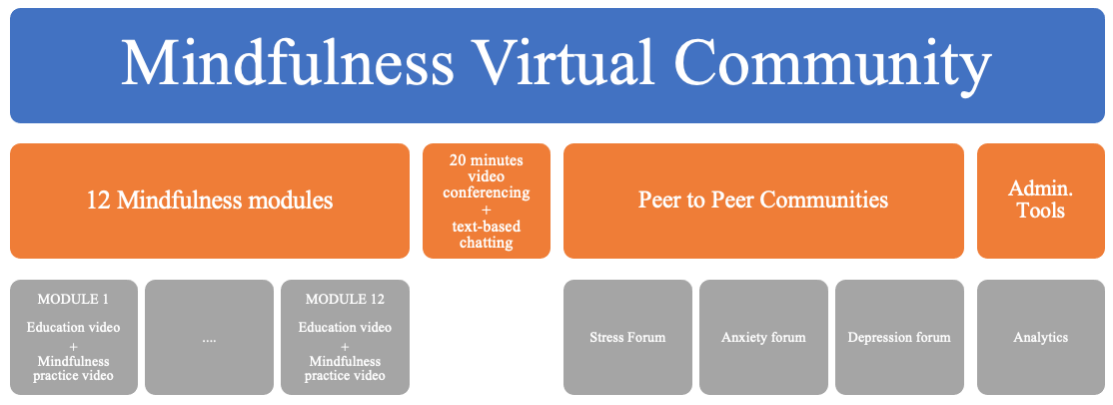
The Mindfulness Virtual Community design.

The MVC intervention was 8 weeks in duration and featured the following: (1) 12 student-specific mental health modules conveyed by online video, (2) 3 anonymous discussion boards dedicated to depression, anxiety, and stress, and (3) an anonymous 20-minute group-based live videoconference led by a moderator (with a master’s degree in psychology), during which students raised and discussed topics covered in the modules (Figure 1).

Each mental health module consisted of 1 educational content video and 1 mindfulness practice video recorded with male and female voices and offered in high- and low-resolution formats (12 videos per module); participants could choose the type of video preferred. The videos were made available for participants 24 hours/day to watch or listen to on internet-linked computers, smartphones, or tablets. The module scripts and resulting audio recordings were created by an investigator with extensive experience as a clinical research psychologist and mindfulness researcher (PR) [29-34]. They drew on mindfulness and CBT principles, with the topics informed by prior focus group study [35,36]. The choice of moving and still images used in the videos involved collaborative work (PR, CE, and FA). The module topics and video durations are presented in Table 1. The role played by the online mindfulness moderator was informed by a pilot study [4].

This study can be characterized as being at stage 2 of the National Institute of Health stage model [37], progressing from a pilot study during which the entire platform was tested. In this study, we continued to test the MVC intervention in a research context with research therapists/providers.

**Table 1:** Topics and duration of modules.

Topics	Education videos (duration in minutes and seconds)	Mindfulness videos (duration in minutes and seconds)
Overcoming stress, anxiety, and depression	7:09	9:00
Mindfulness and being a student	5:18	9:14
Mindfulness for better sleep	4:40	8:13
Thriving in a fast-changing world	7:23	8:23
Healthy intimacy	7:32	9:33
Destigmatization	6:13	9:12
No more procrastination	3:42	10:48
Pain reduction and mindfulness	3:48	9:48
Healthy body image	5:44	9:54
Healthier eating	10:10	9:26
Overcoming trauma	6:01	9:43
Relationships with family and friends	7:49	8:09

New modules were regularly released during the 8-week intervention period. Following release, they were accessible to students for the remaining intervention period. The videoconferences were offered biweekly in three 20-minute evening sessions. The students in the intervention group received email reminders from project staff prior to the release of each module and prior to the live videoconferences. Access to the internet was assumed to be a minor obstacle as the prior focus group study revealed that 94.4% of the students had access to a smartphone and 93.1% had access to laptops or personal home computers. All participants had free internet access on campus and nearly all reported internet access through smartphones and/or laptop computers [38].

The MVC was constructed in partnership with an industry partner (ForaHealthyme Inc) and designed to be a virtual environment supportive of personal mindfulness practice and related CBT self-help. It facilitated mutual help interactions between participants, and between participants and the moderator. The two categories of users were students and health professionals (who moderated the discussion board dialogues and led the live videoconferences). All users used a login and a password to gain access.

Once logged in, each student could do the following: (1) access the educational and mindfulness video modules, (2) access 3 peer-to-peer discussion boards, 1 for each of the 3 mental health conditions targeted by the RCT (anxiety, stress, and depression), (3) notify the moderator about any message posting that represented a problem to the student (eg, online bullying), (4) access a calendar to book an upcoming videoconference, (5) access a virtual “room” that allowed videoconferencing (camera and microphone being off as default) and private text-based chatting with the moderator, and (6) a resource page with contact information for various social and health services.

Once the moderator logged in, they had access to the same options as the students, as well as several additional features: the ability to delete any message on the discussion boards deemed potentially distressing to other users, the ability to populate the calendar with dates and times for upcoming videoconference sessions, the ability to start a videoconference session (camera turned on by default), and the ability to respond privately to incoming text messages in the videoconferencing virtual “room.”

The moderator had weekly supervision sessions with the team psychologist (PR) to optimize responses to the videoconferences and submitted weekly written reports (without individual names) about topics raised by students and responses to them. The content of the modules and the platform structure remained unchanged during the 8-week intervention; the name of the university that received the research grant and the name of the partnering information technology company appeared on the main page of the platform.

### Study Outcomes and Measures Used

The primary outcomes were depression, anxiety, and perceived symptoms of stress. Depression symptoms were assessed with the PHQ-9 [38], where each item is rated on a 0-3 scale and total scores range from 0 to 27 (0-4 indicates minimal/subclinical depression, 5-9 indicates mild depression, 10-14 moderate, 15-19 moderately severe, and ≥20 indicates severe depression). Anxiety was measured using the 21-item BAI [39], where each item is rated on a 0-3 scale and the total score range is 0 to 63 (0-7 indicates a minimal anxiety level, 8-15 a mild anxiety level, 16-25 a moderate anxiety level, and 26-63 a severe anxiety level). For the measurement of stress, we used the 10-item PSS [40], where each item is rated on a 0-4 scale, and the total score range is 0 to 40 (scores of 0-13 indicate mild levels of stress, 14-26 moderate, and 27-40 high). The secondary outcomes were quality of life, life satisfaction, and mindfulness. We used the 16-item Quality of Life Scale (QOLS) [41]; each item is rated on a scale from 1 to 7 and the total score ranges from 16 to 112. Student life satisfaction was measured using the 6-item Brief Multidimensional Students’ Life Satisfaction Scale-Peabody Treatment Progress Battery (BMSLSS-PTPB) [42]; each item is rated on a scale from 1 to 5, and item scores are averaged together to give a total score that ranges from 1 to 5. The level of mindfulness was measured by the 24-item FFMQ-SF [43]; each item is rated on a scale from 1 to 5, and the total score range is 24 to 120. The subscales in the FFMQ-SF are nonreactivity to inner experience (5 items), observing (4 items), acting with awareness (5 items), describing (5 items), and nonjudging of inner experience (5 items). We assessed each of the scales for internal consistency within the T1 and T2 data sets, and Cronbach α ranged from .87 and .90 for PHQ-9; .94 and .94 for BAI; .88 and .90 for PSS; and .85 and .84 for FFMQ-SF” at T1 and T2 respectively. Participants also completed a sociodemographic questionnaire at T1 that inquired about age, gender, birth country, years lived in Canada, first language, relationship status, and ethnic heritage.

All outcomes and other variables were measured by the self-report questionnaires at T1 and T2. The primary RCT outcomes were depression, anxiety, and perceived stress, while mindfulness was measured as a secondary outcome. It was hypothesized that symptom scores for depression, anxiety, and stress at T2 would be significantly lower in the MVC group compared with the WLC group, and that mindfulness scores at T2 would be significantly higher in the MVC group (compared with WLC). The outcomes were measured with the following validated scales: PHQ-9 [40]; BAI [41], PSS [42], and FFMQ-SF [43]. Participants also completed a sociodemographic questionnaire at T1.

### Sample Size and Statistical Analysis

The sample size was calculated for 80% power and 5% type I error to detect a standardized effect size of 0.5 or larger. The required sample size was 63 students per arm. Our recruitment goal was 80 participants per arm, assuming an attrition rate of ~20%.

Descriptive statistics for demographic and psychological characteristics were calculated at baseline, and potential between-group differences were assessed using chi-square tests of independence for categorical variables, and *t* tests for numeric variables. To evaluate the impact of the intervention on study outcomes (ie, depression, anxiety, perceived stress, and mindfulness), generalized estimating equations with an AR (1) covariance structure were employed. Outcome evaluations were performed on both an intention-to-treat (ITT) and per-protocol (PP) basis, with the group × time interaction indicating a between-group change in study outcomes. As the missing data were minimal (<10% overall) and considered to be missing at random, multiple imputations were used to estimate missing observations. For all outcomes, effect sizes are presented for between-group comparisons at follow-up, evaluated by Cohen *d* and calculated as difference between MVC and WLC means divided by their pooled standard deviations. Cohen d effect size for between-group and within-group (repeated measures) comparisons are calculated according to procedures outlined in Lakens [44].

## Results

### Participants

Study participants were 154 undergraduate university students randomized to MVC (n=76) or WLC (n=78) conditions. Of the 154 participants with complete baseline assessments, 7 participants (9.2%) within the MVC and 1 (1.3%) WLC participant dropped out of the study without completing follow-up assessments. As indicated in Table 2, there were no statistically significant between-group differences at baseline in age, gender, country of birth, first language, relationship status, and ethnicity, indicating similar proportions of demographic characteristics across the MVC and WLC groups. Similarly, there were no statistically significant between-group differences in health status and access to private mental health services, with 74.7% of participants across both groups indicating good/very good/excellent health, and 60.4% of participants (across both groups) indicating no access to private mental health services. In addition, there were no significant between-group differences in hours spent at work (paid and unpaid) and in physical activity. In relation to psychological characteristics, no statistically significant between-group differences were detected at baseline between MVC and WLC scores on PHQ-9 (*P*=.88), BAI (*P*=.86), PSS (*P*=.60), and FFMQ-SF (*P*=.44), and in the following subscales: nonreactivity (*P*=.87), observing (*P*=.63), acting with awareness (*P*=.56), describing (*P*=.40), and nonjudgment (*P*=.44).

**Table 2:** Participants’ demographic characteristics at baseline.

Demographic characteristics		All (n=154)	Mindfulness Virtual Community (n=76)	Wait-list control (n=78)	*P* value
Age (years), mean (SD)^a^		23.10 (8.09)	22.02 (5.52)	24.18 (9.95)	.10
**Gender, n (%)**					
	Male	35 (22.7)	18 (23.7)	17 (21.8)	.96
	Female	117 (76.0)	57 (75.0)	60 (76.9)	
	Other	2 (1.3)	1 (1.3)	1 (1.3)	
**Country of birth, n (%)**					
	Canada	91 (59.1)	46 (60.5)	45 (57.7)	.72
	Other	63 (40.9)	30 (39.5)	33 (42.3)	
Years in Canada, mean (SD)^b^		10.73 (7.05)	11.05 (6.21)	10.42 (7.82)	.73
**First language, n (%)**					
	English	103 (66.9)	50 (65.8)	53 (67.9)	.78
	Other	51 (33.1)	26 (34.2)	25 (32.1)	
**Relationship status, n (%)**					
	Single, not in a relationship	79 (51.3)	40 (52.6)	39 (50.0)	.70
	Single, in a relationship	56 (36.4)	27 (35.5)	29 (37.2)	
	Married or common law	13 (8.4)	5 (6.6)	8 (10.3)	
	Divorced, separated, widowed, or other	6 (3.9)	4 (5.3)	2 (2.6)	
**Ethnicity, n (%)**					
	White	40 (26.0)	17 (22.4)	23 (29.5)	.69
	Black	25 (16.2)	11 (14.5)	14 (17.9)	
	South Asian	37 (24.0)	21 (27.6)	16 (20.5)	
	Other	35 (22.7)	19 (25.0)	16 (20.5)	
	Multiple ethnicities	17 (11.0)	8 (10.5)	9 (11.5)	
**Self-rated health, n (%)**					
	Poor or fair	39 (25.3)	23 (30.3)	16 (20.5)	.38
	Good	60 (39.0)	28 (36.8)	32 (41.0)	
	Very good or excellent	55 (35.7)	25 (32.9)	30 (38.5)	
**Access to private mental health care, n (%)**					
	Yes	61 (39.6)	31 (40.8)	30 (38.5)	.77
	No	93 (60.4)	45 (59.2)	48 (61.5)	
**Weekly hours of activities, mean (SD)**					
	Paid work	7.78 (10.60)	7.76 (9.22)	7.81 (11.84)	.98
	Unpaid work (including volunteer work)	2.87 (5.42)	2.89 (5.81)	2.85 (5.04)	.96
	Vigorous physical activities^c^	1.68 (1.87)	1.75 (1.76)	1.62 (1.97)	.66
**Patient Health Questionnaire score, mean (SD)**		9.60 (6.14)	9.53 (5.83)	9.67 (6.50)	.88
	0-9	77 (50.0)	39 (51.3)	38 (48.7)	.74
	≥10	77 (50.0)	37 (48.7)	40 (51.3)	
**Beck Anxiety Inventory score, mean (SD)**		16.88 (13.32)	16.68 (13.56)	17.06 (13.17)	.86
	0-21 (low)	100 (65.0)	49 (64.5)	51 (65.4)	.69
	22-35 (moderate)	35 (22.7)	16 (21.0)	19 (24.4)	
	≥36 (high)	19 (12.3)	11 (14.5)	8 (10.2)	
**Perceived Stress Scale score, mean (SD)**		21.30 (7.66)	21.63 (7.72)	20.99 (7.63)	.60
	0-13 (low)	25 (16.2)	11 (14.5)	14 (17.9)	.74
	14-26 (moderate)	90 (58.4)	44 (57.9)	46 (59.0)	
	27-40 (high)	39 (25.3)	21 (27.6)	18 (23.1)	
Five-Facet Mindfulness Questionnaire score, mean (SD)		74.15 (12.42)	73.36 (11.22)	74.92 (13.52)	.44

^a^Based on n=152 participants (missing data on n=2).

^b^Based on n=63 participants born outside of Canada.

^c^Based on n=151 participants (missing data on n=3).

### Outcome Evaluations

Table 3 shows the means and standard deviations for study outcomes at baseline and at 8-week follow-up for the MVC and WLC groups. Table 3 further includes effect sizes for the mean difference between MVC and WLC at 8-week follow up. The PHQ-9 and the FFMQ-SF exhibited negligible between-group differences at 8 weeks, while the between-group effect sizes for the BAI, PSS, and FFMQ-SF nonjudgment subscale were in the small range (*d*=0.20-0.24).

**Table 3:** Descriptive statistics for depression, anxiety, stress, and mindfulness scores.

Outcomes		Intention to treat			Per protocol		
		Mindfulness Virtual Community, mean (SD)	Wait-list control, mean (SD)	Cohen *d*	Mindfulness Virtual Community, mean (SD)	Wait-list control, mean (SD)	Cohen *d*
		n=76	n=78		n=69	n=77	
**Patient Health Questionnaire-9**							
	Baseline	9.67 (6.50)	9.53 (5.83)	N/A^a^	9.80 (6.63)	9.42 (5.78)	N/A
	8 weeks	7.76 (6.12)	8.05 (6.25)	0.05	7.81 (6.41)	8.05 (6.30)	0.04
**Beck Anxiety Inventory**							
	Baseline	16.68 (13.56)	17.06 (13.17)	N/A	16.91 (13.56)	16.91 (13.19)	N/A
	8 weeks	12.15 (10.50)	14.58 (12.29)	0.21	12.29 (10.84)	14.61 (12.37)	0.20
**Perceived Stress Scale**							
	Baseline	21.63 (7.72)	20.99 (7.63)	N/A	21.52 (7.74)	20.99 (7.68)	N/A
	8 weeks	18.44 (7.51)	20.11 (7.83)	0.22	18.28 (7.82)	20.12 (7.88)	0.24
**Five Facets Mindfulness Questionnaire-Short Form**							
	Baseline	73.35 (11.22)	74.92 (13.52)	N/A	73.22 (11.10)	74.92 (13.61)	N/A
	8 weeks	75.65 (10.08)	75.89 (12.49)	0.02	75.62 (10.58)	75.89 (12.57)	0.02
**Five Facets Mindfulness Questionnaire-Short Form, Nonreactivity subscale**							
	Baseline	14.33 (3.79)	14.22 (4.31)	N/A	14.20 (3.77)	14.20 (4.34)	N/A
	8 weeks	14.65 (3.26)	14.69 (3.58)	0.01	14.67 (3.39)	14.68 (3.60)	0.003
**Five Facets Mindfulness Questionnaire-Short Form, Observing subscale**							
	Baseline	13.38 (3.31)	13.65 (3.64)	N/A	13.38 (3.33)	13.69 (3.65)	N/A
	8 weeks	13.46 (3.18)	13.90 (3.43)	0.13	13.45 (3.33)	13.90 (3.45)	0.13
**Five Facets Mindfulness Questionnaire-Short Form, Acting with Awareness subscale**							
	Baseline	15.54 (4.12)	15.92 (4.08)	N/A	15.72 (4.10)	15.92 (4.11)	N/A
	8 weeks	16.10 (3.87)	16.39 (4.08)	0.07	16.09 (4.05)	16.40 (4.11)	0.08
**Five Facets Mindfulness Questionnaire-Short Form, Describing subscale**							
	Baseline	16.32 (4.16)	16.89 (4.15)	N/A	16.22 (4.30)	16.90 (4.17)	N/A
	8 weeks	16.92 (3.51)	17.19 (4.26)	0.07	16.90 (3.68)	17.18 (4.29)	0.07
**Five Facets Mindfulness Questionnaire-Short Form, Nonjudgement subscale**							
	Baseline	13.79 (3.28)	14.24 (3.99)	N/A	13.69 (3.28)	14.22 (4.01)	N/A
	8 weeks	14.52 (3.48)	13.72 (3.69)	0.22	14.52 (3.65)	13.73 (3.71)	0.22

^a^N/A: not applicable.

Table 4 presents the results for ITT and PP models on the impact of the intervention on PHQ-9, BAI, PSS, and FFMQ-SF scores, including FFMQ-SF subscale scores. Except for PSS, there were no statistically significant reductions compared to WLC. For PSS, compared to the WLC group, the MVC group had a statistically significant reduction with both ITT and PP analytic approaches (ITT: β=–2.31, *P*=.03; PP: β=–2.38, *P*=.03). When compared to the WLC group, the MVC group also exhibited a slight increase in the FFMQ-SF nonjudgment subscale (ITT: β=1.25, *P*=.06; PP: β=1.31, *P*=.06).

**Table 4:** Generalized estimating equations for depression, anxiety, stress, and mindfulness outcomes from baseline to 8 weeks.

Outcomes		Intention to treat	*P* value	Per protocol	*P* value
		Mean (SE), 95% CI		Mean (SE), 95% CI	
**Patient Health Questionnaire-9**					
	Intercept	9.53 (0.66), 8.24 to 10.81	<.001	9.42 (0.65), 8.13 to 10.70	<.001
	Time	–1.47 (0.63), –2.70 to –0.25	.02	–1.37 (0.63), –2.59 to –0.14	.03
	Group	0.14 (0.99), –1.79 to 2.08	.88	0.38 (1.03), –1.63 to 2.40	.71
	Group × Time	–0.44 (0.95), –2.31 to 1.43	.64	–0.62 (0.99), –2.57 to 1.32	.53
**Beck Anxiety Inventory**					
	Intercept	17.06 (1.48), 14.16 to 19.97	<.001	16.91 (1.49), 13.98 to 19.84	<.001
	Time	–2.48 (1.51), –5.44 to 0.49	.10	–2.30 (1.52), –5.28 to 0.68	.13
	Group	–0.38 (2.14), –4.58 to 3.82	.86	0.004 (2.20), –4.31 to 4.32	.99
	Group × Time	–2.06 (2.02), –6.03 to 1.92	.31	–2.32 (2.09), –6.42 to 1.77	.27
**Perceived Stress Scale**					
	Intercept	20.99 (0.86), 19.31 to 22.67	<.001	20.99 (0.87), 19.28 to 22.69	<.001
	Time	–0.88 (0.69), –2.24 to 0.48	.20	–0.87 (0.70), –2.24 to 0.50	.22
	Group	0.64 (1.23), –1.77 to 3.05	.60	0.53 (1.27), –1.95 to 3.02	.67
	Group × Time	–2.31 (1.06), –4.39 to –0.24	.03	–2.38 (1.10), –4.54 to –0.22	.03
**Five Facets Mindfulness Questionnaire-Short Form**					
	Intercept	74.92 (1.52), 71.94 to 77.90	<.001	74.92 (1.54), 71.90 to 77.94	<.001
	Time	0.97 (0.99), –0.97 to 2.90	.33	0.97 (1.00), –0.99 to 2.93	.33
	Group	–1.57 (1.99), –5.46 to 2.33	.43	–1.70 (2.03), –5.69 to 2.28	.40
	Group × Time	1.33 (1.69), –1.99 to 4.64	.43	1.44 (1.75), –1.99 to 4.87	.41
**Five Facets Mindfulness Questionnaire-Short Form, Nonreactivity subscale**					
	Intercept	14.22 (0.49), 13.27 to 15.17	<.001	14.20 (0.49), 13.23 to 15.16	<.001
	Time	0.47 (0.38), –0.28 to 1.22	.22	0.48 (0.39), –0.28 to 1.24	.21
	Group	0.11 (0.65), –1.16 to 1.39	.86	0.008 (0.66), –1.30 to 1.32	.99
	Group × Time	–0.15 (0.64), –1.40 to 1.10	.81	–0.02 (0.67), –1.32 to 1.29	.98
**Five Facets Mindfulness Questionnaire-Short Form, Observing subscale**					
	Intercept	13.65 (0.41), 12.85 to 14.46	<.001	13.69 (0.41), 12.88 to 14.50	<.001
	Time	0.25 (0.32), –0.39 to 0.88	.45	0.21 (0.32), –0.43 to 0.85	.52
	Group	–0.27 (0.56), –1.36 to 0.82	.63	–0.31 (0.57), –1.44 to 0.81	.59
	Group × Time	–0.16 (0.53), –1.20 to 0.88	.76	–0.14 (0.56), –1.23 to 0.95	.80
**Five Facets Mindfulness Questionnaire-Short Form, Acting with Awareness subscale**					
	Intercept	15.92 (0.46), 15.02 to 16.82	<.001	15.92 (0.46), 15.01 to 16.83	<.001
	Time	0.47 (0.43), –0.38 to 1.31	.28	0.48 (0.44), –0.38 to 1.33	.27
	Group	–0.38 (0.66), –1.67 to 0.91	.56	–0.20 (0.68), –1.52 to 1.13	.77
	Group × Time	0.09 (0.66), –1.21 to 1.39	.89	–0.11 (0.68), –1.46 to 1.23)	.87
**Five Facets Mindfulness Questionnaire-Short Form, Describing subscale**					
	Intercept	16.89 (0.47), 15.97 to 17.80	<.001	16.90 (0.47), 15.97 to 17.82	<.001
	Time	0.30 (0.34), –0.36 to 0.97	.37	0.28 (0.34), –0.39 to 0.95	.41
	Group	–0.57 (0.66), –1.87 to 0.73	.39	–0.68 (0.70), –2.05 to 0.69	.33
	Group × Time	0.31 (0.50), –0.68 to 1.29	.54	0.40 (0.52), –0.63 to 1.43	.45
**Five Facets Mindfulness Questionnaire-Short Form, Nonjudgement subscale**					
	Intercept	14.24 (0.45), 13.36 to 15.12	<.001	14.22 (0.45), 13.33 to 15.11	<.001
	Time	–0.52 (0.40), –1.29 to 0.26	.18	–0.49 (0.40), –1.27 to 0.30	.22
	Group	–0.45 (0.58), –1.60 to 0.69	.44	–0.53 (0.60), –1.70 to 0.65	.38
	Group × Time	1.25 (0.65), –0.03 to 2.53	.06	1.31 (0.69), –0.04 to 2.66	.06

Tables 5 and 6 show additional analyses that focused on discerning which subgroups were affected more or less by the intervention (given varying baseline depression, anxiety, and perceived stress levels). Across 3 levels of depressive symptoms per PHQ-9 categories (ie, mild, moderate, and severe symptom levels), intervention group reductions were greater than those of the control group. An exception was the moderately severe category, where the greater effect size observed was in the controls. We have highlighted the Cohen *d* effect sizes to facilitate comparisons. The moderately severe category has modest effect size differences.

When similar analyses involved female-male comparisons, the effect sizes and mean reductions were greater in females across mild, moderate, moderately severe, and severe categories. Reductions in cell size are noted, particularly for the male data, as the female study sample far exceeded the male sample (Tables 7 and 8).

**Table 5:** Analysis of Patient Health Questionnaire-9 subgroups: intervention.

Patient Health Questionnaire-9, intervention	Minimal (0-4)	Mild (5-9)	Moderate (10-14)	Moderately severe (15-19)	Severe (20-27)
	N=20	N=19	N=20	N=11	N=6
Mean (T1/T2)	2.20/4.86	6.84/5.41	11.95/8.79	16.54/12.70	23.33/12.33
Mean difference, Cohen *d*	2.66, 0.64	–1.43, 0.50	–3.16, 0.67	–3.84, 0.55	–11.0, 2.18

**Table 6:** Analysis of Patient Health Questionnaire-9 subgroups: control.

Patient Health Questionnaire-9, wait-list control	Minimal (0-4)	Mild (5-9)	Moderate (10-14)	Moderately severe (15-19)	Severe (20-27)
	N=17	N=21	N=26	N=10	N=4
Mean (T1/T2)	1.82/2.17	7.19/6.52	11.61/10.53	16.90/12.41	22.50/14.00
Mean difference, Cohen *d*	0.35, 0.20	–0.67, 0.19	–1.08, 0.29	–4.49, 0.68	–8.5, 1.52

**Table 7:** Analysis of Patient Health Questionnaire-9 subgroups: male.

Patient Health Questionnaire-9, intervention, females	Minimal (0-4)	Mild (5-9)	Moderate (10-14)	Moderately severe (15-19)	Severe (20-27)
	N=14	N=16	N=13	N=9	N=5
Mean (T1/T2)	2.36/3.37	7.00/5.31	11.92/7.82	16.44/12.19	22.60/11.00
Mean difference, Cohen *d*	1.01, 0.44	–1.69, 0.56	–4.1, 1.24	–4.25, 0.58	–11.6, 3.07

**Table 8:** Analysis of Patient Health Questionnaire-9 subgroups: female.

Patient Health Questionnaire-9, intervention, males	Minimal (0-4)	Mild (5-9)	Moderate (10-14)	Moderately severe (15-19)	Severe (20-27)
	N=5	N=3	N=7	N=2	N=1
Mean (T1/T2)	2.00/5.00	6.00/5.93	12.00/10.57	17.00/15.00	27.00/19.00
Mean difference, Cohen *d*	3.00, 0.97	–0.07, 0.33	–1.43, 0.18	–2.00, 0.37	–8.00, N/A

In established BAI categories (ie, low, moderate, and severe symptoms) intervention group reductions were less than control reductions in both low and high symptom categories, while in the moderate symptom category, intervention reductions were greater (Tables 9 and 10). When analyses focused on comparing females with males, the effect sizes and mean reductions were greater in females across moderate and high categories, while the low category results can be characterized as equal. There were reductions in cell size due to the female-male comparisons (Tables 11 and 12).

**Table 9:** Analysis of Beck Anxiety Inventory categories: intervention.

Beck Anxiety Inventory, intervention	Low (0-21 )	Moderate (22-35)	High (≥36)
	N=49	N=16	N=11
Mean (T1/T2)	8.06/8.41	26.00/17.29	41.54/21.34
Mean difference, Cohen *d*	0.35, 0.05	–8.71, 1.27	–20.21, 1.72

**Table 10:** Analysis of Beck Anxiety Inventory categories: control.

Beck Anxiety Inventory, wait-list controls	Low (0-21 )	Moderate (22-35)	High (≥36)
	N=51	N=19	N=8
Mean (T1/T2)	8.90/11.02	28.05/20.77	43.00/22.62
Mean difference, Cohen *d*	2.12, 0.22	–7.28, 0.93	–20.38, 2.31

**Table 11:** Analysis of Beck Anxiety Inventory categories: female.

Beck Anxiety Inventory, intervention, females	Low (0-21 )	Moderate (22-35)	High (≥36)
	N=36	N=14	N=7
Mean (T1/T2)	8.42/8.32	26.14/16.54	43.14/21.53
Mean difference, Cohen *d*	–0.10, 0.01	–9.60, 1.38	–21.61, 1.84

**Table 12:** Analysis of Beck Anxiety Inventory categories: male.

Beck Anxiety Inventory, intervention, males	Low (0-21 )	Moderate (22-35)	High (≥36)
	N=12	N=2	N=4
Mean (T1/T2)	7.50/8.05	25.0/22.5	38.75/21.00
Mean difference, Cohen *d*	0.55, 0.07	–2.50, 0.16	–17.75, 0.77

In perceived stress, intervention and control comparisons demonstrated, at the moderate and high stress levels, greater reductions in the intervention group with approximately equal reductions observed at the low level (Tables 13 and 14). When analyses focused on female-male comparisons, in the moderate and high levels, females demonstrated more change than males, while in the low group females had a negative intervention effect (their stress increased) while males had no intervention effects (ie, neither positive or negative; Tables 15 and 16).

**Table 13:** Analysis of Perceived Stress Scale categories: intervention.

Perceived Stress Scale, intervention	Low (0-13)	Moderate (14-26)	High (27-40)
	N=11	N=44	N=21
Mean (T1/T2)	9.55/11.09	20.25/17.93	30.86/23.36
Mean difference, Cohen *d*	1.54, 0.26	–2.32, 0.40	–7.5, 1.59

**Table 14:** Analysis of Perceived Stress Scale categories: control.

Perceived Stress Scale, wait-list controls	Low (0-13)	Moderate (14-26)	High (27-40)
	N=14	N=46	N=18
Mean (T1/T2)	9.14/10.64	20.78/20.57	30.72/26.28
Mean difference, Cohen *d*	1.5, 0.28	–0.21, 0.04	–4.44, 0.88

**Table 15:** Analysis of Perceived Stress Scale categories: female.

Perceived Stress Scale, intervention, female	Low (0-13)	Moderate (14-26)	High (27-40)
	N=8	N=33	N=16
Mean (T1/T2)	8.87/11.0	19.97/17.55	31.25/23.41
Mean difference, Cohen *d*	2.13, 0.50	–2.42, 0.42	–7.84, 1.77

**Table 16:** Analysis of Perceived Stress Scale categories: male.

Perceived Stress Scale, intervention, males	Low (0-13)	Moderate (14-26)	High (27-40)
	N=3	N=11	N=4
Mean (T1/T2)	11.33/11.33	21.09/19.06	30.0/21.25
Mean difference, Cohen *d*	0.00, 0	–2.03, 0.33	–8.75, 1.33

Altogether, females gained more benefit than males and intervention subjects benefitted more than controls. Gains were most evident in depression and perceived stress reductions. Anxiety results (BAI outcomes) were more variable, likely reflecting the immediate anxiety reduction effects in controls with the suspension of expected exams and assignments.

## Discussion

### Principal Findings

This study was undertaken at a labor strike–affected university, following identical procedures described in two prior studies at the same site, conducted when no crises existed [4,5]. Accordingly, participant responses to the previous RCTs are usefully compared to responses in this study (referred to as “Study 3” below).

In the previously reported RCTs [4,5], significant between-group differences were observed in depression (PHQ-9), anxiety (BAI), quality of life (QOLS), and mindfulness (FFMQ-SF). In the first RCT, significant between-group differences were additionally found in perceived stress (PSS). In this study, a significant between-group difference was only found in the PSS (*P*=.03), while a marginal (*P*=.06) difference was observed in the Nonjudgment subscale of FFMQ-SF.

Despite the limited between-group differences in this disrupted-campus study, significant group × time effects suggested that the MVC intervention had a positive impact on students. However, while in Study 1 and 2 the WLC depression scores (PHQ-9) [4,5] increased over time (Time 2>Time 1; perhaps due to anticipating increasingly demanding academic tasks), in Study 3, the WLC subjects had substantially reduced depression scores (Time 1<Time 2, by –1.4 in mean raw score). As the intervention within-group reductions were nearly equivalent across studies 1, 2, and 3, the significant between-group differences absent in Study 3 appeared related to the self-reported depression reductions (between Time 1 and Time 2) in WLC subjects.

Cross-study differences were also observable in the BAI indices, where in Study 1 and 2, WLC scores increased over time (Time 2>Time 1), whereas in Study 3 the WLC scores decreased over time (Time 2<Time 1) by a mean (raw) scale score of –2.38. Once again, the within-group reductions were nearly equivalent across studies 1, 2, and 3 (–4.2, –4.8, and –4.5, respectively) indicating that reductions in the between-group differences in Study 3 were related to anxiety reductions in the WLC group.

Other interesting cross-study differences were observable in PSS indices where significant between-group differences were found in Study 1 and Study 3, but not in Study 2. In Study 1 and 3, the WLC group scores were nearly equivalent (Time 1 and Time 2) as were the reductions achieved in the intervention group (Study 1: –3.10, Study 2: –3.19, in raw scores).

One interpretation of such study comparisons is that students experienced the crisis as reducing academic pressure, with the result of them experiencing less distress (represented in lower mean WLC group depression and anxiety scores). While the lowered distress was likely temporary (outcomes were assessed 8 weeks after baseline, and prior to resumed course activity), the pattern might have also affected motivations regarding participation in the intervention. If so, this would have contributed to the lack of significant between-group differences in depression and anxiety in this study.

It is notable that while there were no significant between-group differences previously observed in Study 2 for the PSS, there was a significant difference observed in this study (Study 3) and Study 1. It seems that while the burdens of depression and anxiety in the WLC group were reduced in Study 3, there was not an equivalent reduction of the immediate stress represented in PSS items. Several PSS items refer to “unexpected happenings beyond control, anger and irritation due to loss of control,” and positive control (a reversed item) over “important things.” Such items reflect the lost control that can occur during a campus strike. Thus, significant between-group differences favoring the intervention could have occurred because intervention participants felt positive intervention effects related to the disruption caused by the strike. Why PSS differences were found in Study 1 is an interesting question for speculation. Notably, tense, publicized negotiations preceded the strike, during the period when Study 1 was undertaken. Students might have been aware of the increasing strike probability, with that awareness represented on the PSS intervention-attributable differences.

If the academic disruption was experienced as a temporary reduction in distressing academic pressures, the “strike” reaction is congruent with focus group findings preceding the 3 RCTs, which helped prioritize intervention topics [37,38]. Specifically, from focus group findings, “procrastination” was reported as a prevalent student problem. Their transcript responses referred to seeking and finding internet distraction while ignoring difficult academic tasks. A frequently described experience was that initially brief divergences consumed more time than planned and resulted in delays in “getting down to work,” causing schoolwork to suffer and anxieties to increase.

These descriptions appear similar to the internet-based problems described in interviews with members of the I-Gen (generation) cohort [42]. For example, given normative engagement in more internet-based interactions, only a few keystrokes separate tasks of high consequence from stress-reducing “escapes” (using computers and smartphones).

While few motivational obstacles accompany the transition from “work” to entertainment, the return to committed work is more difficult. This contrasts with the differences experienced when one physically goes to a library or classroom and has peer interaction. While distracting temptations exist, social reinforcements counteract tendencies to surrender to distractions. The face-to-face shutdown that largely happened around the York University strike led to reduced academic pressures and normative social academic reinforcements.

Approximately 30%-60% of undergraduate students report regular procrastination in studying for exams, writing term papers, and doing weekly readings to the point where performance is compromised [45-49]. The increased stress due to procrastination can manifest as anxiety, irritation, regret, despair, and/or self-blame [45-53].

Procrastination in students can be associated with maladaptive perfectionism [31,32], where students develop perfectionistic attitudes and project the likely disapproval of others. Under these conditions, procrastination serves to control and reduce the anxieties stemming from such dysfunctional attitudes. These perspectives were demonstrated to have validity in previous intervention trials [31,32] and were applied in the MVC intervention content.

In closing, student participants in an 8-week RCT demonstrated behavior changes, although the study site was a strike-affected and disrupted campus. In two previously published RCTs at the same site under normal conditions, with the same intervention, significant between-group differences in depression, anxiety, quality of life, and mindfulness were observed, with significant between-group differences in perceived stress in one RCT (Study 1) but not in the other (Study 2). In the current strike-affected RCT, there was a significant between-group difference in perceived stress but an absence of significant findings regarding depression, anxiety, quality of life, and mindfulness. Further examinations indicated the between-study differences were, to some degree, the result of lower (when compared to the previous trials) depression/anxiety self-report observed in the WLC group rather than positive intervention group changes. One interpretation of these differences was that students experienced the disruption that occurred during the 8-week trial as a reprieve or pressure easing. The RCT participants self-reported being less anxious and depressed within the 8 weeks. On the other hand, the presence of significant between-group differences in perceived stress demonstrated the assistance received from the intervention following the more immediate loss of control due to life disruptions (ie, strikes).

Several study limitations must be taken into account. This was not a single- or double-blinded trial. The study duration of 8 weeks did not allow us to test for longer-term effects (eg, 6 or 12 months), which would have resulted in a more fully assessed crisis effect. A further limitation is the high female preponderance in the control and intervention groups, as more precise future research projects would address gender differences through stratification with larger participant samples from multiple research sites (involving multiple universities and colleges). Missing data was also a limitation, somewhat mitigated by the use of a multiple imputation method. Lastly and importantly, given the marginal between-group differences found in this study in mindfulness self-report (FFMQ-SF), there was no measure of the participants’ mindfulness practice external to platform participation.

### Conclusion

Our results suggest that an 8-week online M-CBT video-based program is an effective intervention for reducing perceived stress among undergraduate university students on a disrupted campus. To some degree, the online M-CBT interventions offer an opportunity to address mental health conditions in postsecondary populations under disrupted campus conditions that might have similarities to COVID-19–related disruptions, which may have resulted in face-to-face academic interactions being suspended.

## Appendix

Multimedia Appendix 1 CONSORT-eHEALTH checklist (V 1.6.1).

## Abbreviations

BAI: Beck Anxiety Inventory
BMSLSS-PTPB: Brief Multidimensional Students’ Life Satisfaction Scale-Peabody Treatment Progress Battery
FFMQ-SF: Five Facets Mindfulness Questionnaire Short Form
ITT: intention to treat
M-CBT: mindfulness-based cognitive behavioral therapy
MVC: Mindfulness Virtual Community
PHQ-9: Patient Health Questionnaire-9
PP: per protocol
PSS: Perceived Stress Scale
QOLS: Quality of Life Scale
RCT: randomized controlled trial
WLC: wait-list control
