# Correction: A Mindfulness-Based Intervention for Student Depression, Anxiety, and Stress: Randomized Controlled Trial

**DOI:** 10.2196/27160

**Published:** 2021-01-25

**Authors:** Paul Ritvo, Farah Ahmad, Christo El Morr, Meysam Pirbaglou, Rahim Moineddin, MVC Team

**Affiliations:** 1 School of Kinesiology and Health Science Faculty of Health York University Toronto, ON Canada; 2 School of Health Policy and Management York University Toronto, ON Canada; 3 Dalla Lana School of Public Health University of Toronto Toronto, ON Canada; 4 York University Toronto, ON Canada

In “A Mindfulness-Based Intervention for Student Depression, Anxiety, and Stress: Randomized Controlled Trial” (JMIR Ment Health 2021;8(1):e23491) the authors noted one error.

This paper was inadvertently published with an equal contribution footnote for the authors *Paul Ritvo, Farah Ahmad, Christo El Morr,* and the group author *MVC Team*. This was incorrect, as only the authors *Paul Ritvo, Farah Ahmad,* and *Christo El Morr* contributed equally. The equal contribution footnote for MVC Team has been removed.

The correction will appear in the online version of the paper on the JMIR Publications website on January 25, 2021, together with the publication of this correction notice. Because this was made after submission to PubMed, PubMed Central, and other full-text repositories, the corrected article has also been resubmitted to those repositories.

