# Supplementary material for: A Mindfulness-Based Intervention for Student Depression, Anxiety, and Stress: Randomized Controlled Trial
Source: JMIR Ment Health. 2021 Jan 11;8(1):e23491. doi: 10.2196/23491 (PMC7833974; doi:10.2196/23491)
Supplement: Multimedia Appendix 1 [file mental_v8i1e23491_app1.pdf]

CONSORT-EHEALTH (V 1.6.1) - Submission/Publication Form

The CONSORT-EHEALTH checklist is intended for authors of randomized trials evaluating web-based and Internet-based applications/interventions, including mobile interventions, electronic games (incl multiplayer games), social media, certain telehealth applications, and other interactive and/or networked electronic applications. Some of the items (e.g. all subitems under item 5 - description of the intervention) may also be applicable for other study designs.

The goal of the CONSORT EHEALTH checklist and guideline is to be
a) a guide for reporting for authors of RCTs,
b) to form a basis for appraisal of an ehealth trial (in terms of validity)

CONSORT-EHEALTH items/subitems are MANDATORY reporting items for studies published in the Journal of Medical Internet Research and other journals / scientific societies endorsing the checklist.

Items numbered 1., 2., 3., 4a., 4b etc are original CONSORT or CONSORT-NPT (non-pharmacologic treatment) items.
Items with Roman numerals (i., ii, iii, iv etc.) are CONSORT-EHEALTH extensions/clarifications.

As the CONSORT-EHEALTH checklist is still considered in a formative stage, we would ask that you also RATE ON A SCALE OF 1-5 how important/useful you feel each item is FOR THE PURPOSE OF THE CHECKLIST and reporting guideline (optional).

Mandatory reporting items are marked with a red *.
In the textboxes, either copy & paste the relevant sections from your manuscript into this form - please include any quotes from your manuscript in QUOTATION MARKS,
or answer directly by providing additional information not in the manuscript, or elaborating on why the item was not relevant for this study.

YOUR ANSWERS WILL BE PUBLISHED AS A SUPPLEMENTARY FILE TO YOUR PUBLICATION IN JMIR AND ARE CONSIDERED PART OF YOUR PUBLICATION (IF ACCEPTED).
Please fill in these questions diligently. Information will not be copyedited, so please use proper spelling and grammar, use correct capitalization, and avoid abbreviations.

DO NOT FORGET TO SAVE AS PDF _AND_ CLICK THE SUBMIT BUTTON SO YOUR ANSWERS ARE IN OUR DATABASE !!!

Citation Suggestion (if you append the pdf as Appendix we suggest to cite this paper in the caption):
Eysenbach G, CONSORT-EHEALTH Group
CONSORT-EHEALTH: Improving and Standardizing Evaluation Reports of Web-based and

Mobile Health Interventions
J Med Internet Res 2011;13(4):e126
URL: http://www.jmir.org/2011/4/e126/
doi: 10.2196/jmir.1923
PMID: 22209829

* Required

Your name *

First Last

Paul Ritvo

Primary Affiliation (short), City, Country *

University of Toronto, Toronto, Canada

York University, Toronto, Canada

Your e-mail address *

abc@gmail.com

pritvo@yorku.ca

Title of your manuscript *

Provide the (draft) title of your manuscript.

A Mindfulness-Based Intervention for Student Depression, Anxiety and Stress: Randomized Controlled Trial

Name of your App/Software/Intervention *

If there is a short and a long/alternate name, write the short name first and add the long name in brackets.

MVC (Mindfulness Virtual Community)

Evaluated Version (if any)

e.g. "V1", "Release 2017-03-01", "Version 2.0.27913"

Your answer

Language(s) *

What language is the intervention/app in? If multiple languages are available, separate by comma (e.g. "English, French")

English

URL of your Intervention Website or App

e.g. a direct link to the mobile app on app in appstore (itunes, Google Play), or URL of the website. If the intervention is a DVD or hardware, you can also link to an Amazon page.

http://www.studentsmentalhealth.com

URL of an image/screenshot (optional)

Your answer

Accessibility *

Can an enduser access the intervention presently?

- ○ access is free and open
- ○ access only for special usergroups, not open
- ○ access is open to everyone, but requires payment/subscription/in-app purchases
- ○ app/intervention no longer accessible
- ◉ Other: access to modules can be discussed with the authors

Primary Medical Indication/Disease/Condition *

e.g. "Stress", "Diabetes", or define the target group in brackets after the condition, e.g. "Autism (Parents of children with)", "Alzheimers (Informal Caregivers of)"

Depression, anxiety and stress symptoms

Primary Outcomes measured in trial *

comma-separated list of primary outcomes reported in the trial

Depression, anxiety and perceived stress

Secondary/other outcomes

Are there any other outcomes the intervention is expected to affect?

Mindfulness (Five Facet Mindfulness Questionnaire

Recommended "Dose" *

What do the instructions for users say on how often the app should be used?

- ( ) Approximately Daily
- ( ) Approximately Weekly
- ( ) Approximately Monthly
- ( ) Approximately Yearly
- ( ) "as needed"
- (x) Other: ad libitum

Approx. Percentage of Users (starters) still using the app as recommended after 3 months *

- ( ) unknown / not evaluated
- ( ) 0-10%
- ( ) 11-20%
- ( ) 21-30%
- ( ) 31-40%
- ( ) 41-50%
- ( ) 51-60%
- ( ) 61-70%
- ( ) 71%-80%
- ( ) 81-90%
- ( ) 91-100%
- (x) Other: Participants did not have access after the 8 week study period

Overall, was the app/intervention effective? *

- [ ] yes: all primary outcomes were significantly better in intervention group vs control
- [x] partly: SOME primary outcomes were significantly better in intervention group vs control
- [ ] no statistically significant difference between control and intervention
- [ ] potentially harmful: control was significantly better than intervention in one or more outcomes
- [ ] inconclusive: more research is needed
- [ ] Other:

Article Preparation Status/Stage *

At which stage in your article preparation are you currently (at the time you fill in this form)

- [ ] not submitted yet - in early draft status
- [ ] not submitted yet - in late draft status, just before submission
- [ ] submitted to a journal but not reviewed yet
- [ ] submitted to a journal and after receiving initial reviewer comments
- [x] submitted to a journal and accepted, but not published yet
- [ ] published
- [ ] Other:

Journal *

If you already know where you will submit this paper (or if it is already submitted), please provide the journal name (if it is not JMIR, provide the journal name under "other")

- ◯ not submitted yet / unclear where I will submit this
- ◯ Journal of Medical Internet Research (JMIR)
- ◯ JMIR mHealth and UHealth
- ◯ JMIR Serious Games
- ◉ JMIR Mental Health
- ◯ JMIR Public Health
- ◯ JMIR Formative Research
- ◯ Other JMIR sister journal
- ◯ Other:

Is this a full powered effectiveness trial or a pilot/feasibility trial? *

- ◯ Pilot/feasibility
- ◉ Fully powered

Manuscript tracking number *

If this is a JMIR submission, please provide the manuscript tracking number under "other" (The ms tracking number can be found in the submission acknowledgement email, or when you login as author in JMIR. If the paper is already published in JMIR, then the ms tracking number is the four-digit number at the end of the DOI, to be found at the bottom of each published article in JMIR)

- ◯ no ms number (yet) / not (yet) submitted to / published in JMIR
- ◉ Other: 23491

TITLE AND ABSTRACT

1a) TITLE: Identification as a randomized trial in the title

1a) Does your paper address CONSORT item 1a? *

I.e does the title contain the phrase "Randomized Controlled Trial"? (if not, explain the reason under "other")

- (selected) yes
- ( ) Other:

1a-i) Identify the mode of delivery in the title

Identify the mode of delivery. Preferably use "web-based" and/or "mobile" and/or "electronic game" in the title. Avoid ambiguous terms like "online", "virtual", "interactive". Use "Internet-based" only if Intervention includes non-web-based Internet components (e.g. email), use "computer-based" or "electronic" only if offline products are used. Use "virtual" only in the context of "virtual reality" (3-D worlds). Use "online" only in the context of "online support groups". Complement or substitute product names with broader terms for the class of products (such as "mobile" or "smart phone" instead of "iphone"), especially if the application runs on different platforms.

	1	2	3	4	5	
subitem not at all important	○	○	○	○	●	essential

Clear selection

Does your paper address subitem 1a-i? *

Copy and paste relevant sections from manuscript title (include quotes in quotation marks "like this" to indicate direct quotes from your manuscript), or elaborate on this item by providing additional information not in the ms, or briefly explain why the item is not applicable/relevant for your study

Web-based

1a-ii) Non-web-based components or important co-interventions in title

Mention non-web-based components or important co-interventions in title, if any (e.g., “with telephone support”).

	1	2	3	4	5	
subitem not at all important	○	◉	○	○	○	essential

Clear selection

Does your paper address subitem 1a-ii?

Copy and paste relevant sections from manuscript title (include quotes in quotation marks "like this" to indicate direct quotes from your manuscript), or elaborate on this item by providing additional information not in the ms, or briefly explain why the item is not applicable/relevant for your study

All co-interventions were delivered via the web-based platform

1a-iii) Primary condition or target group in the title

Mention primary condition or target group in the title, if any (e.g., “for children with Type I Diabetes”) Example: A Web-based and Mobile Intervention with Telephone Support for Children with Type I Diabetes: Randomized Controlled Trial

	1	2	3	4	5	
subitem not at all important	○	○	○	○	◉	essential

Clear selection

Does your paper address subitem 1a-iii? *

Copy and paste relevant sections from manuscript title (include quotes in quotation marks "like this" to indicate direct quotes from your manuscript), or elaborate on this item by providing additional information not in the ms, or briefly explain why the item is not applicable/relevant for your study

University students on symptoms of stress, anxiety and depression

1b) ABSTRACT: Structured summary of trial design, methods, results, and conclusions

NPT extension: Description of experimental treatment, comparator, care providers, centers, and blinding status.

1b-i) Key features/functionalities/components of the intervention and comparator in the METHODS section of the ABSTRACT

Mention key features/functionalities/components of the intervention and comparator in the abstract. If possible, also mention theories and principles used for designing the site. Keep in mind the needs of systematic reviewers and indexers by including important synonyms. (Note: Only report in the abstract what the main paper is reporting. If this information is missing from the main body of text, consider adding it)

	1	2	3	4	5	
subitem not at all important	○	○	○	○	◉	essential

Clear selection

Does your paper address subitem 1b-i? *

Copy and paste relevant sections from the manuscript abstract (include quotes in quotation marks "like this" to indicate direct quotes from your manuscript), or elaborate on this item by providing additional information not in the ms, or briefly explain why the item is not applicable/relevant for your study

"In this parallel-arm RCT, n = 154 students were randomly allocated to an 8 week MVC intervention (n = 76) or a wait-list control (WLC) condition (n = 78). The MVC intervention included the following: 1) education and mindfulness video modules, 2) anonymous peer-to-peer discussion and 3) anonymous group-based, professionally guided, 20-minute video conferences."

1b-ii) Level of human involvement in the METHODS section of the ABSTRACT

Clarify the level of human involvement in the abstract, e.g., use phrases like "fully automated" vs. "therapist/nurse/care provider/physician-assisted" (mention number and expertise of providers involved, if any). (Note: Only report in the abstract what the main paper is reporting. If this information is missing from the main body of text, consider adding it)

	1	2	3	4	5	
subitem not at all important	○	○	◉	○	○	essential

Clear selection

Does your paper address subitem 1b-ii?

Copy and paste relevant sections from the manuscript abstract (include quotes in quotation marks "like this" to indicate direct quotes from your manuscript), or elaborate on this item by providing additional information not in the ms, or briefly explain why the item is not applicable/relevant for your study

"The MVC intervention included the following: 1) educational and mindfulness video modules, 2) anonymous peer-to-peer discussions, and 3) anonymous, group-based, professionally guided, 20-minute video conferences."

1b-iii) Open vs. closed, web-based (self-assessment) vs. face-to-face assessments in the METHODS section of the ABSTRACT

Mention how participants were recruited (online vs. offline), e.g., from an open access website or from a clinic or a closed online user group (closed usergroup trial), and clarify if this was a purely web-based trial, or there were face-to-face components (as part of the intervention or for assessment). Clearly say if outcomes were self-assessed through questionnaires (as common in web-based trials). Note: In traditional offline trials, an open trial (open-label trial) is a type of clinical trial in which both the researchers and participants know which treatment is being administered. To avoid confusion, use "blinded" or "unblinded" to indicated the level of blinding instead of "open", as "open" in web-based trials usually refers to "open access" (i.e. participants can self-enrol). (Note: Only report in the abstract what the main paper is reporting. If this information is missing from the main body of text, consider adding it)

	1	2	3	4	5	
subitem not at all important	○	○	○	○	◉	essential

Clear selection

Does your paper address subitem 1b-iii?

Copy and paste relevant sections from the manuscript abstract (include quotes in quotation marks "like this" to indicate direct quotes from your manuscript), or elaborate on this item by providing additional information not in the ms, or briefly explain why the item is not applicable/relevant for your study

"In this parallel-arm RCT, n = 154 student were randomly allocated to an 8-week MVC intervention (n = 76) or a wait-list control (WLC) condition (n = 78)."
We could not use the exact wording of "blinded" in the Abstract as students and research assists were blinded only until the stage of consent provision.

1b-iv) RESULTS section in abstract must contain use data

Report number of participants enrolled/assessed in each group, the use/uptake of the intervention (e.g., attrition/adherence metrics, use over time, number of logins etc.), in addition to primary/secondary outcomes. (Note: Only report in the abstract what the main paper is reporting. If this information is missing from the main body of text, consider adding it)

	1	2	3	4	5	
subitem not at all important	○	○	○	○	◉	essential

Clear selection

Does your paper address subitem 1b-iv?

Copy and paste relevant sections from the manuscript abstract (include quotes in quotation marks "like this" to indicate direct quotes from your manuscript), or elaborate on this item by providing additional information not in the ms, or briefly explain why the item is not applicable/relevant for your study

"Participants (n = 154) included 35 males and 117 females with a mean age of 23.1 years. There were no statistically significant differences at baseline between the MVC and WLC groups on demographics and psychological characteristics, indicating similar demographic and psychological characteristics, indicating similar demographic and psychological characteristics across the two groups. Results under both Intention to Treat (ITT) and Per Protocol (PP) approaches indicated that there no statistically significant differences in PhQ-9 (ITT:β= -0.44, P = .64; PP: β= -0.62, P =.53), BAI (ITT:β= -2.06, P=.31; PP: β= -2.32, P = .27) and FFMQ-SF (ITT: β= 1.33, P=.43; PP: β= 1.44, P=.41) compared to WLC. There was a significant difference for the PSS (ITT: β= -2.31, P=.03; PP: β= -2.38, P = .03)"

1b-v) CONCLUSIONS/DISCUSSION in abstract for negative trials

Conclusions/Discussions in abstract for negative trials: Discuss the primary outcome - if the trial is negative (primary outcome not changed), and the intervention was not used, discuss whether negative results are attributable to lack of uptake and discuss reasons. (Note: Only report in the abstract what the main paper is reporting. If this information is missing from the main body of text, consider adding it)

	1	2	3	4	5	
subitem not at all important	○	○	○	◉	○	essential

Clear selection

Does your paper address subitem 1b-v?

Copy and paste relevant sections from the manuscript abstract (include quotes in quotation marks "like this" to indicate direct quotes from your manuscript), or elaborate on this item by providing additional information not in the ms, or briefly explain why the item is not applicable/relevant for your study

"This study was undertaken at a labor strike–affected university, following identical procedures described in two prior studies at the same site, conducted when no crises existed [4,5]. Accordingly, participant responses to the previous RCTs are usefully compared to responses in this study (referred to as “Study 3” below).
In the previously reported RCTs [4,5], significant between-group differences were observed in depression (PHQ-9), anxiety (BAI), quality of life (QOLS), and mindfulness (FFMQ-SF). In the first RCT, significant between-group differences were additionally found in perceived stress (PSS). In this study, a significant between-group difference was only found in the PSS (P=.03), while a marginal (P=.06) difference was observed in the Nonjudgment subscale of FFMQ-SF. Despite the limited between-group differences in this disrupted-campus study, significant group × time effects suggested that the MVC intervention had a positive impact on students. However, while in Study 1 and 2 the WLC depression scores (PHQ-9) [4,5] increased over time (Time 2>Time 1; perhaps due to anticipating increasingly demanding academic tasks), in Study 3, the WLC subjects had substantially reduced depression scores (Time 1<Time 2, by –1.4 in mean raw score). As the intervention within-group reductions were nearly equivalent across studies 1, 2, and 3, the significant between-group differences absent in Study 3 appeared related to the self-reported depression reductions (between Time 1 and Time 2) in WLC subjects. "

INTRODUCTION

2a) In INTRODUCTION: Scientific background and explanation of rationale

2a-i) Problem and the type of system/solution

Describe the problem and the type of system/solution that is object of the study: intended as stand-alone intervention vs. incorporated in broader health care program? Intended for a particular patient population? Goals of the intervention, e.g., being more cost-effective to other interventions, replace or complement other solutions? (Note: Details about the intervention are provided in “Methods” under 5)

	1	2	3	4	5	
subitem not at all important	○	○	○	○	◉	essential

Clear selection

Does your paper address subitem 2a-i? *

Copy and paste relevant sections from the manuscript (include quotes in quotation marks "like this" to indicate direct quotes from your manuscript), or elaborate on this item by providing additional information not in the ms, or briefly explain why the item is not applicable/relevant for your study

University students are experiencing higher levels of distress and mental health disorders than before. In addressing mental health needs, web-based interventions have shown increasing promise in overcoming geographic distances and high student-to-counselor ratios, leading to the potential for wider implementation. The Mindfulness Virtual Community (MVC) program, a web-based program, guided by mindfulness and cognitive behavioral therapy principles, is among efforts aimed at effectively and efficiently reducing symptoms of depression, anxiety, and perceived stress in students.
"The impact of COVID-19 and other disruptions on education, generally, and secondary education, specifically, can have major student mental health effects. COVID-19–related shutdowns of face-to-face high school education have been associated with significant student dropout rates in multiple cities in the United States, as high proportions of students, engaged face-to-face, failed to connect online (13%) [1]. With the escalating infection rates of both spring and fall 2020, many university courses that were initiated in face-to-face formats were completed online [2,3], with considerable uncertainties about the fall 2020 semester and the impact of COVID-19 outbreaks on campus life generally.
However, the response of students to other campus crises can provide useful data in anticipating the effects of other disruptions. In 2018, during a 4-year project assessing the effects of mindfulness-based cognitive behavioral therapy (M-CBT), York University was impacted by a faculty and teaching assistant strike. Most students respected picket lines and did not attend classes until the strike resolved, which occurred after the end of the usual calendar-defined semester. Despite the strike, study recruitment and intervention testing proceeded. Here, we report the results of that randomized controlled trial (RCT).
The RCT undertaken was identical to two prior studies [4,5] on the same campus site under non-crisis conditions. As the studies were undertaken months apart, there were minimal environmental differences, other than seasonal change. In the initial study [4], 113 students (mean age 24.8 years) participated in an 8-week online M-CBT program and significant between-group benefits were found on measures of depression (Patient Health Questionnaire-9 [PHQ-9]), anxiety (Beck Anxiety Inventory [BAI]), quality of life (Quality of Life Scale [QOLS]), and mindfulness (Five Facet Mindfulness Questionnaire - Short Form [FFMQ-SF]), favoring the intervention group (MVC) compared to wait-list controls (WLC). In the follow-up study [5], 159 students (mean age 22.5 years) participated in the same 8-[illegible]

week online M-CBT program and significant between-group differences were again found on identical measures of depression (PHQ-9), anxiety (BAI), mindfulness (FFMQ-SF), and quality of life in intervention participants, compared to wait-list controls. These two demonstrations of intervention efficacy prepared us to investigate how the campus crisis may have affected student responses.
All three studies were motivated by the rising prevalence of mental health disorders, including depression and anxiety, among college students worldwide, prior to the COVID-19 pandemic [6]. In the United States, for example, analyses of college data show that mental health disorders are among the top 5 diagnostic categories seen at college health services and are responsible for the highest number of visits (4.93) per student [7]. Multiple US studies have suggested there is an increasing prevalence of mental health disorders, especially depression and anxiety, among undergraduate college and university students [7-14].
Additionally, of concern for online researchers, there are possible links between decreased youth mental health and increased online activity (ie, “screen time”) [15-17]. Strong arguments and reliable data support the perspective that online activities, particularly social media engagement, have unhealthy impacts for some youth populations [15-17]. Disruptions of regular face-to-face classes at universities and high schools may have elevated levels of unhealthy “screen time” for select populations. Accordingly, it is worthwhile to ascertain what changes might have occurred when university students experiencing a disruption that likely increased time spent online used an online mental health intervention."

2a-ii) Scientific background, rationale: What is known about the (type of) system

Scientific background, rationale: What is known about the (type of) system that is the object of the study (be sure to discuss the use of similar systems for other conditions/diagnoses, if appropiate), motivation for the study, i.e. what are the reasons for and what is the context for this specific study, from which stakeholder viewpoint is the study performed, potential impact of findings [2]. Briefly justify the choice of the comparator.

	1	2	3	4	5	
subitem not at all important	○	○	○	◉	○	essential

Clear selection

Does your paper address subitem 2a-ii? *

Copy and paste relevant sections from the manuscript (include quotes in quotation marks "like this" to indicate direct quotes from your manuscript), or elaborate on this item by providing additional information not in the ms, or briefly explain why the item is not applicable/relevant for your study

"Accordingly, we developed a web-delivered M-CBT program (aimed at creating a Mindfulness Virtual Community, or MVC) to reduce depression, anxiety, and stress in university students and, as mentioned above, previously assessed in two prior RCTs targeting Canadian university students that indicated efficacy [4,5].

2b) In INTRODUCTION: Specific objectives or hypotheses

Does your paper address CONSORT subitem 2b? *

Copy and paste relevant sections from the manuscript (include quotes in quotation marks "like this" to indicate direct quotes from your manuscript), or elaborate on this item by providing additional information not in the ms, or briefly explain why the item is not applicable/relevant for your study

"The impact of COVID-19 and other disruptions on education, generally, and secondary education, specifically, can have major student mental health effects. COVID-19–related shutdowns of face-to-face high school education have been associated with significant student dropout rates in multiple cities in the United States, as high proportions of students, engaged face-to-face, failed to connect online (13%) [1]. With the escalating infection rates of both spring and fall 2020, many university courses that were initiated in face-to-face formats were completed online [2,3], with considerable uncertainties about the fall 2020 semester and the impact of COVID-19 outbreaks on campus life generally.
However, the response of students to other campus crises can provide useful data in anticipating the effects of other disruptions. In 2018, during a 4-year project assessing the effects of mindfulness-based cognitive behavioral therapy (M-CBT), York University was impacted by a faculty and teaching assistant strike. Most students respected picket lines and did not attend classes until the strike resolved, which occurred after the end of the usual calendar-defined semester. Despite the strike, study recruitment and intervention testing proceeded. Here, we report the results of that randomized controlled trial (RCT)."
"The RCT undertaken was identical to two prior studies [4,5] on the same campus site under non-crisis conditions. As the studies were undertaken months apart, there were minimal environmental differences, other than seasonal change. In the initial study [4], 113 students (mean age 24.8 years) participated in an 8-week online M-CBT program and significant between-group benefits were found on measures of depression (Patient Health Questionnaire-9 [PHQ-9]), anxiety (Beck Anxiety Inventory [BAI]), quality of life (Quality of Life Scale [QOLS]), and mindfulness (Five Facet Mindfulness Questionnaire - Short Form [FFMQ-SF]), favoring the intervention group (MVC) compared to wait-list controls (WLC). In the follow-up study [5], 159 students (mean age 22.5 years) participated in the same 8-week online M-CBT program and significant between-group differences were again found on identical measures of depression (PHQ-9), anxiety (BAI), mindfulness (FFMQ-SF), and quality of life in intervention participants, compared to wait-list controls. These two demonstrations of intervention efficacy prepared us to investigate how the campus crisis may have affected student responses.
All three studies were motivated by the rising prevalence of mental health disorders, including depression and anxiety, among college students worldwide, prior to the COVID-19 pandemic [6]. In the United States, for example, analyses of [illegible]

college data show that mental health disorders are among the top 5 diagnostic categories seen at college health services and are responsible for the highest number of visits (4.93) per student [7]. Multiple US studies have suggested there is an increasing prevalence of mental health disorders, especially depression and anxiety, among undergraduate college and university students [7-14]."
"Additionally, of concern for online researchers, there are possible links between decreased youth mental health and increased online activity (ie, “screen time”) [15-17]. Strong arguments and reliable data support the perspective that online activities, particularly social media engagement, have unhealthy impacts for some youth populations [15-17]. Disruptions of regular face-to-face classes at universities and high schools may have elevated levels of unhealthy “screen time” for select populations. Accordingly, it is worthwhile to ascertain what changes might have occurred when university students experiencing a disruption that likely increased time spent online used an online mental health intervention."
"Accordingly, we developed a web-delivered M-CBT program (aimed at creating a Mindfulness Virtual Community, or MVC) to reduce depression, anxiety, and stress in university students and, as mentioned above, previously assessed in two prior RCTs targeting Canadian university students that indicated efficacy [4,5]. "

METHODS

3a) Description of trial design (such as parallel, factorial) including allocation ratio

Does your paper address CONSORT subitem 3a? *

Copy and paste relevant sections from the manuscript (include quotes in quotation marks "like this" to indicate direct quotes from your manuscript), or elaborate on this item by providing additional information not in the ms, or briefly explain why the item is not applicable/relevant for your study

This study was a two-arm parallel-design RCT comparing the web-based Mindfulness Virtual Community program to a wait-list control group. The Human Participant Research Committee at York University provided research and ethics approval for RCT (Certificate number: e2016-345)

3b) Important changes to methods after trial commencement (such as eligibility criteria), with reasons

Does your paper address CONSORT subitem 3b? *

Copy and paste relevant sections from the manuscript (include quotes in quotation marks "like this" to indicate direct quotes from your manuscript), or elaborate on this item by providing additional information not in the ms, or briefly explain why the item is not applicable/relevant for your study

There were no changes in the eligibility criteria or recruitment methods.

3b-i) Bug fixes, Downtimes, Content Changes

Bug fixes, Downtimes, Content Changes: ehealth systems are often dynamic systems. A description of changes to methods therefore also includes important changes made on the intervention or comparator during the trial (e.g., major bug fixes or changes in the functionality or content) (5-iii) and other “unexpected events” that may have influenced study design such as staff changes, system failures/downtimes, etc. [2].

	1	2	3	4	5	
subitem not at all important	○	○	○	◉	○	essential

Clear selection

Does your paper address subitem 3b-i?

Copy and paste relevant sections from the manuscript (include quotes in quotation marks "like this" to indicate direct quotes from your manuscript), or elaborate on this item by providing additional information not in the ms, or briefly explain why the item is not applicable/relevant for your study

"The content of the modules and the platform structure remained unchanged during the 8-week intervention."

4a) Eligibility criteria for participants

Does your paper address CONSORT subitem 4a? *

Copy and paste relevant sections from the manuscript (include quotes in quotation marks "like this" to indicate direct quotes from your manuscript), or elaborate on this item by providing additional information not in the ms, or briefly explain why the item is not applicable/relevant for your study

"Eligibility criteria were applied to recruit actively enrolled undergraduates aged ≥18 years, with English-language fluency and self-reported confidence in completing the study. Students were excluded if they reported substance abuse or episodes of psychosis during the month prior to the trial.
The study was advertised using study posters, class announcements, and email invitations via list servs of student associations in the Faculties of Health and Liberal Arts. Interested students contacted the research staff via email or phone and were screened for student registration, substance abuse, and indications of psychoses. If abuse or psychotic behaviors “interfered in routine life within the last month,” students were excluded and provided with a list of accessible mental health resources. In addition, a registered clinical psychologist could be contacted directly if there was a perceived need for interim mental health counseling."

4a-i) Computer / Internet literacy

Computer / Internet literacy is often an implicit “de facto” eligibility criterion - this should be explicitly clarified.

	1	2	3	4	5	
subitem not at all important	○	○	○	◉	○	essential

Clear selection

Does your paper address subitem 4a-i?

Copy and paste relevant sections from the manuscript (include quotes in quotation marks "like this" to indicate direct quotes from your manuscript), or elaborate on this item by providing additional information not in the ms, or briefly explain why the item is not applicable/relevant for your study

Average university-student levels of computer and internet literacy were assumed. If there were participant reported difficulties, these were addressed in personal, confidential consultations with the study coordinators.

4a-ii) Open vs. closed, web-based vs. face-to-face assessments:

Open vs. closed, web-based vs. face-to-face assessments: Mention how participants were recruited (online vs. offline), e.g., from an open access website or from a clinic, and clarify if this was a purely web-based trial, or there were face-to-face components (as part of the intervention or for assessment), i.e., to what degree got the study team to know the participant. In online-only trials, clarify if participants were quasi-anonymous and whether having multiple identities was possible or whether technical or logistical measures (e.g., cookies, email confirmation, phone calls) were used to detect/prevent these.

	1	2	3	4	5	
subitem not at all important	○	○	○	○	◉	essential

Clear selection

Does your paper address subitem 4a-ii? *

Copy and paste relevant sections from the manuscript (include quotes in quotation marks "like this" to indicate direct quotes from your manuscript), or elaborate on this item by providing additional information not in the ms, or briefly explain why the item is not applicable/relevant for your study

"The study was advertised using study posters, class announcements, and email invitations via list servs of student associations in the Faculties of Health and Liberal Arts. Interested students contacted the research staff via email or phone and were screened for student registration, substance abuse, and indications of psychoses. If abuse or psychotic behaviors “interfered in routine life within the last month,” students were excluded and provided with a list of accessible mental health resources. In addition, a registered clinical psychologist could be contacted directly if there was a perceived need for interim mental health counseling.
Eligible and willing students received detailed in-person information about the study and provided informed written consent. Participants had the option to receive an honorarium of Can $50 (US $39) or 2% in course grade (for professors who gave permission for this option) or three credits (equivalent to 2% course grade) in the Undergraduate Research Participation Pool (URPP) of the Department of Psychology. Each participant also received a resource list that included information about health and social services on campus and in the community (eg, the 24/7 “Good to Talk” helpline for postsecondary students in Ontario). Our protocol included a safety mechanism whereby participants were asked verbally and on the consent form to contact the research staff if they felt distress during the trial period so that “limited counselling with a clinical psychologist could be arranged, if needed.”
"Each participant in the MVC group received a unique ID and a temporary password; participants changed passwords after their first login, while IDs remained the same to reduce the potential of multiple accounts or identities.
Participants in all groups completed online questionnaires at baseline (T1) and 8 weeks (T2)."

4a-iii) Information giving during recruitment

Information given during recruitment. Specify how participants were briefed for recruitment and in the informed consent procedures (e.g., publish the informed consent documentation as appendix, see also item X26), as this information may have an effect on user self-selection, user expectation and may also bias results.

	1	2	3	4	5	
subitem not at all important	○	○	○	◉	○	essential

Clear selection

Does your paper address subitem 4a-iii?

Copy and paste relevant sections from the manuscript (include quotes in quotation marks "like this" to indicate direct quotes from your manuscript), or elaborate on this item by providing additional information not in the ms, or briefly explain why the item is not applicable/relevant for your study

"Eligible and willing students received detailed in-person information about the study and provided informed written consent. Participants had the option to receive an honorarium of Can $50 (US $39) or 2% in course grade (for professors who gave permission for this option) or three credits (equivalent to 2% course grade) in the Undergraduate Research Participation Pool (URPP) of the Department of Psychology. Each participant also received a resource list that included information about health and social services on campus and in the community (eg, the 24/7 “Good to Talk” helpline for postsecondary students in Ontario). Our protocol included a safety mechanism whereby participants were asked verbally and on the consent form to contact the research staff if they felt distress during the trial period so that “limited counselling with a clinical psychologist could be arranged, if needed.” No such requests arose during the reported study period."

4b) Settings and locations where the data were collected

Does your paper address CONSORT subitem 4b? *

Copy and paste relevant sections from the manuscript (include quotes in quotation marks "like this" to indicate direct quotes from your manuscript), or elaborate on this item by providing additional information not in the ms, or briefly explain why the item is not applicable/relevant for your study

Participants in all groups completed the online questionnaires at baseline (T1) and 8 weeks (T2).

4b-i) Report if outcomes were (self-)assessed through online questionnaires

Clearly report if outcomes were (self-)assessed through online questionnaires (as common in web-based trials) or otherwise.

	1	2	3	4	5	
subitem not at all important	○	○	○	●	○	essential

Clear selection

Does your paper address subitem 4b-i? *

Copy and paste relevant sections from the manuscript (include quotes in quotation marks "like this" to indicate direct quotes from your manuscript), or elaborate on this item by providing additional information not in the ms, or briefly explain why the item is not applicable/relevant for your study

"Participants in all groups completed the online questionnaires at baseline (T1) and 8 weeks (T2)."

4b-ii) Report how institutional affiliations are displayed

Report how institutional affiliations are displayed to potential participants [on ehealth media], as affiliations with prestigious hospitals or universities may affect volunteer rates, use, and reactions with regards to an intervention.(Not a required item – describe only if this may bias results)

	1	2	3	4	5	
subitem not at all important	○	○	○	●	○	essential

Clear selection

Does your paper address subitem 4b-ii?

Copy and paste relevant sections from the manuscript (include quotes in quotation marks "like this" to indicate direct quotes from your manuscript), or elaborate on this item by providing additional information not in the ms, or briefly explain why the item is not applicable/relevant for your study

The name of the university that received the research grant and the name of the partnering information technology company appeared on the main page of the platform.

5) The interventions for each group with sufficient details to allow replication, including how and when they were actually administered

5-i) Mention names, credential, affiliations of the developers, sponsors, and owners

Mention names, credential, affiliations of the developers, sponsors, and owners [6] (if authors/evaluators are owners or developer of the software, this needs to be declared in a “Conflict of interest” section or mentioned elsewhere in the manuscript).

	1	2	3	4	5	
subitem not at all important	○	○	○	●	○	essential

Clear selection

Does your paper address subitem 5-i?

Copy and paste relevant sections from the manuscript (include quotes in quotation marks "like this" to indicate direct quotes from your manuscript), or elaborate on this item by providing additional information not in the ms, or briefly explain why the item is not applicable/relevant for your study

In the intervention sub-section: The module scripts and resulting audio recordings were created by an investigator with extensive experience as a clinical research psychologist and mindfulness researcher (PR) [29-34]. They drew on mindfulness and CBT principles, with the topics informed by prior focus group study [35,36]. The choice of moving and still images used in the videos involved collaborative work (PR, CE, and FA).

In the Conflicts of Interest section:
"It is the understanding of the university and researchers that the Project Intellectual Property belongs to CEM, FA and PR. The industry partner, ForaHealthyMe.com owns all rights and titles to the copyrights of any computer source code software that was developed out of this research project."

In the Acknowledgements section:
" The authors acknowledge the contribution of all students who gave their valuable time to participate in the study. We thank ForaHealthyMe.com, Inc. as an industry partner who provided great support. The work reported was funded by the Canadian Institutes for Health Research (CIHR) eHealth Innovations Partnership Program Grant (eHIPP; Grant No.EH1-143553). The project's principal investigators are CE (Nominal PI), FA and PR."

5-ii) Describe the history/development process

Describe the history/development process of the application and previous formative evaluations (e.g., focus groups, usability testing), as these will have an impact on adoption/use rates and help with interpreting results.

	1	2	3	4	5	
subitem not at all important	○	○	○	◉	○	essential

Clear selection

Does your paper address subitem 5-ii?

Copy and paste relevant sections from the manuscript (include quotes in quotation marks "like this" to indicate direct quotes from your manuscript), or elaborate on this item by providing additional information not in the ms, or briefly explain why the item is not applicable/relevant for your study

"The module scripts and resulting audio recordings were created by an investigator with extensive experience as a clinical research psychologist and mindfulness researcher (PR) [29-34]. They drew on mindfulness and CBT principles, with the topics informed by prior focus group study [35,36]. "

5-iii) Revisions and updating

Revisions and updating. Clearly mention the date and/or version number of the application/intervention (and comparator, if applicable) evaluated, or describe whether the intervention underwent major changes during the evaluation process, or whether the development and/or content was “frozen” during the trial. Describe dynamic components such as news feeds or changing content which may have an impact on the replicability of the intervention (for unexpected events see item 3b).

	1	2	3	4	5	
subitem not at all important	○	○	○	●	○	essential

Clear selection

Does your paper address subitem 5-iii?

Copy and paste relevant sections from the manuscript (include quotes in quotation marks "like this" to indicate direct quotes from your manuscript), or elaborate on this item by providing additional information not in the ms, or briefly explain why the item is not applicable/relevant for your study

"The content of the modules and the platform structure remained unchanged during the 8-week intervention."

5-iv) Quality assurance methods

Provide information on quality assurance methods to ensure accuracy and quality of information provided [1], if applicable.

	1	2	3	4	5	
subitem not at all important	○	○	○	○	◉	essential

Clear selection

Does your paper address subitem 5-iv?

Copy and paste relevant sections from the manuscript (include quotes in quotation marks "like this" to indicate direct quotes from your manuscript), or elaborate on this item by providing additional information not in the ms, or briefly explain why the item is not applicable/relevant for your study

We used the parallel-arm RCT design and registered the trial before collecting data and initiating intervention delivery. We provide comprehensive details about intervention development and the focus groups that informed content and design. We used validated measures for primary and secondary outcomes. The group allocation was generated by an off-site biostatistician and it was sealed in opaque envelopes/ The participants and research assistants were blind to the allocation until after the written consent.

Each participant in the MVC intervention group received a unique ID and a temporary password; participants changed the passwords after their first login, while IDs remained the same until after the written consent.

5-v) Ensure replicability by publishing the source code, and/or providing screenshots/screen-capture video, and/or providing flowcharts of the algorithms used

Ensure replicability by publishing the source code, and/or providing screenshots/screen-capture video, and/or providing flowcharts of the algorithms used. Replicability (i.e., other researchers should in principle be able to replicate the study) is a hallmark of scientific reporting.

	1	2	3	4	5	
subitem not at all important	○	○	○	●	○	essential

Clear selection

Does your paper address subitem 5-v?

Copy and paste relevant sections from the manuscript (include quotes in quotation marks "like this" to indicate direct quotes from your manuscript), or elaborate on this item by providing additional information not in the ms, or briefly explain why the item is not applicable/relevant for your study

Figure 1 presents the intervention components. Details on the module content are provided in Table 1 for the 12 topics and the durations of each video.

5-vi) Digital preservation

Digital preservation: Provide the URL of the application, but as the intervention is likely to change or disappear over the course of the years; also make sure the intervention is archived (Internet Archive, webcitation.org, and/or publishing the source code or screenshots/videos alongside the article). As pages behind login screens cannot be archived, consider creating demo pages which are accessible without login.

	1	2	3	4	5	
subitem not at all important	○	○	○	●	○	essential

Clear selection

Does your paper address subitem 5-vi?

Copy and paste relevant sections from the manuscript (include quotes in quotation marks "like this" to indicate direct quotes from your manuscript), or elaborate on this item by providing additional information not in the ms, or briefly explain why the item is not applicable/relevant for your study

Screen short is provided in a previous (linked) publication.

5-vii) Access

Access: Describe how participants accessed the application, in what setting/context, if they had to pay (or were paid) or not, whether they had to be a member of specific group. If known, describe how participants obtained “access to the platform and Internet” [1]. To ensure access for editors/reviewers/readers, consider to provide a “backdoor” login account or demo mode for reviewers/readers to explore the application (also important for archiving purposes, see vi).

	1	2	3	4	5	
subitem not at all important	○	○	○	◉	○	essential

Clear selection

Does your paper address subitem 5-vii? *

Copy and paste relevant sections from the manuscript (include quotes in quotation marks "like this" to indicate direct quotes from your manuscript), or elaborate on this item by providing additional information not in the ms, or briefly explain why the item is not applicable/relevant for your study

"Eligibility criteria were applied to recruit actively enrolled undergraduates aged ≥18 years, with English-language fluency and self-reported confidence in completing the study. Students were excluded if they reported substance abuse or episodes of psychosis during the month prior to the trial.
The study was advertised using study posters, class announcements, and email invitations via list servs of student associations in the Faculties of Health and Liberal Arts. Interested students contacted the research staff via email or phone and were screened for student registration, substance abuse, and indications of psychoses. If abuse or psychotic behaviors “interfered in routine life within the last month,” students were excluded and provided with a list of accessible mental health resources. In addition, a registered clinical psychologist could be contacted directly if there was a perceived need for interim mental health counseling.
Eligible and willing students received detailed in-person information about the study and provided informed written consent. Participants had the option to receive an honorarium of Can $50 (US $39) or 2% in course grade (for professors who gave permission for this option) or three credits (equivalent to 2% course grade) in the Undergraduate Research Participation Pool (URPP) of the Department of Psychology. Each participant also received a resource list that included information about health and social services on campus and in the community (eg, the 24/7 “Good to Talk” helpline for postsecondary students in Ontario). Our protocol included a safety mechanism whereby participants were asked verbally and on the consent form to contact the research staff if they felt distress during the trial period so that “limited counselling with a clinical psychologist could be arranged, if needed.” No such requests arose during the reported study period.
A sample of 480 students (160 students per group) was recruited over 3 semesters (Fall 2017, Winter 2018, and Fall 2018). The 3 samples were not combined due to the campus environment differences related to the 3-month strike in Winter 2018. Here, we report on a sample of n=154 students in a two-arm RCT."

5-viii) Mode of delivery, features/functionalities/components of the intervention and comparator, and the theoretical framework

Describe mode of delivery, features/functionalities/components of the intervention and comparator, and the theoretical framework [6] used to design them (instructional strategy [1], behaviour change techniques, persuasive features, etc., see e.g., [7, 8] for terminology). This includes an in-depth description of the content (including where it is coming from and who developed it) [1],” whether [and how] it is tailored to individual circumstances and allows users to track their progress and receive feedback” [6]. This also includes a description of communication delivery channels and – if computer-mediated communication is a component – whether communication was synchronous or asynchronous [6]. It also includes information on presentation strategies [1], including page design principles, average amount of text on pages, presence of hyperlinks to other resources, etc. [1].

	1	2	3	4	5	
subitem not at all important	○	○	○	●	○	essential

Clear selection

Does your paper address subitem 5-viii? *

Copy and paste relevant sections from the manuscript (include quotes in quotation marks "like this" to indicate direct quotes from your manuscript), or elaborate on this item by providing additional information not in the ms, or briefly explain why the item is not applicable/relevant for your study

"The MVC intervention was 8 weeks in duration and featured the following: (1) 12 student-specific mental health modules conveyed by online video, (2) 3 anonymous discussion boards dedicated to depression, anxiety, and stress, and (3) an anonymous 20-minute group-based live videoconference led by a moderator (with a master’s degree in psychology), during which students raised and discussed topics covered in the modules (Figure 1).
Each mental health module consisted of 1 educational content video and 1 mindfulness practice video recorded with male and female voices and offered in high- and low-resolution formats (12 videos per module); participants could choose the type of video preferred. The videos were made available for participants 24 hours/day to watch or listen to on internet-linked computers, smartphones, or tablets. The module scripts and resulting audio recordings were created by an investigator with extensive experience as a clinical research psychologist and mindfulness researcher (PR) [29-34]. They drew on mindfulness and CBT principles, with the topics informed by prior focus group study [35,36]. The choice of moving and still images used in the videos involved collaborative work (PR, CE, and FA). The module topics and video durations are presented in Table 1."

5-ix) Describe use parameters

Describe use parameters (e.g., intended “doses” and optimal timing for use). Clarify what instructions or recommendations were given to the user, e.g., regarding timing, frequency, heaviness of use, if any, or was the intervention used ad libitum.

	1	2	3	4	5	
subitem not at all important	○	○	○	◉	○	essential

Clear selection

Does your paper address subitem 5-ix?

Copy and paste relevant sections from the manuscript (include quotes in quotation marks "like this" to indicate direct quotes from your manuscript), or elaborate on this item by providing additional information not in the ms, or briefly explain why the item is not applicable/relevant for your study

"Participants were encouraged to use the platform as often as desired."

5-x) Clarify the level of human involvement

Clarify the level of human involvement (care providers or health professionals, also technical assistance) in the e-intervention or as co-intervention (detail number and expertise of professionals involved, if any, as well as “type of assistance offered, the timing and frequency of the support, how it is initiated, and the medium by which the assistance is delivered”. It may be necessary to distinguish between the level of human involvement required for the trial, and the level of human involvement required for a routine application outside of a RCT setting (discuss under item 21 – generalizability).

	1	2	3	4	5	
subitem not at all important	○	○	○	◉	○	essential

Clear selection

Does your paper address subitem 5-x?

Copy and paste relevant sections from the manuscript (include quotes in quotation marks "like this" to indicate direct quotes from your manuscript), or elaborate on this item by providing additional information not in the ms, or briefly explain why the item is not applicable/relevant for your study

"The MVC intervention was 8 weeks in duration and featured the following: (1) 12 student-specific mental health modules conveyed by online video, (2) 3 anonymous discussion boards dedicated to depression, anxiety, and stress, and (3) an anonymous 20-minute group-based live videoconference led by a moderator (with a master’s degree in psychology), during which students raised and discussed topics covered in the modules (Figure 1). "
"The MVC was constructed in partnership with an industry partner (ForaHealthyme Inc) and designed to be a virtual environment supportive of personal mindfulness practice and related CBT self-help. It facilitated mutual help interactions between participants, and between participants and the moderator. The two categories of users were students and health professionals (who moderated the discussion board dialogues and led the live videoconferences). All users used a login and a password to gain access."
" The video conferences were offered biweekly in three 20 minute evening sessions. The students in the intervention group received email reminders from project staff prior to the release of each module and prior to the live video conferences."

5-xi) Report any prompts/reminders used

Report any prompts/reminders used: Clarify if there were prompts (letters, emails, phone calls, SMS) to use the application, what triggered them, frequency etc. It may be necessary to distinguish between the level of prompts/reminders required for the trial, and the level of prompts/reminders for a routine application outside of a RCT setting (discuss under item 21 – generalizability).

	1	2	3	4	5	
subitem not at all important	○	○	○	◉	○	essential

Clear selection

Does your paper address subitem 5-xi? *

Copy and paste relevant sections from the manuscript (include quotes in quotation marks "like this" to indicate direct quotes from your manuscript), or elaborate on this item by providing additional information not in the ms, or briefly explain why the item is not applicable/relevant for your study

The student in the intervention group received email reminders from project staff prior to the release of each module and prior to the live videoconferences.

5-xii) Describe any co-interventions (incl. training/support)

Describe any co-interventions (incl. training/support): Clearly state any interventions that are provided in addition to the targeted eHealth intervention, as ehealth intervention may not be designed as stand-alone intervention. This includes training sessions and support [1]. It may be necessary to distinguish between the level of training required for the trial, and the level of training for a routine application outside of a RCT setting (discuss under item 21 – generalizability.

	1	2	3	4	5	
subitem not at all important	○	○	○	●	○	essential

Clear selection

Does your paper address subitem 5-xii? *

Copy and paste relevant sections from the manuscript (include quotes in quotation marks "like this" to indicate direct quotes from your manuscript), or elaborate on this item by providing additional information not in the ms, or briefly explain why the item is not applicable/relevant for your study

No other co-interventions were provided.

6a) Completely defined pre-specified primary and secondary outcome measures, including how and when they were assessed

Does your paper address CONSORT subitem 6a? *

Copy and paste relevant sections from the manuscript (include quotes in quotation marks "like this" to indicate direct quotes from your manuscript), or elaborate on this item by providing additional information not in the ms, or briefly explain why the item is not applicable/relevant for your study

The primary outcomes were depression, anxiety, and perceived symptoms of stress. Depression symptoms were assessed with the PHQ-9 [38], where each item is rated on a 0-3 scale and total scores range from 0 to 27 (0-4 indicates minimal/subclinical depression, 5-9 indicates mild depression, 10-14 moderate, 15-19 moderately severe, and ≥20 indicates severe depression). Anxiety was measured using the 21-item BAI [39], where each item is rated on a 0-3 scale and the total score range is 0 to 63 (0-7 indicates a minimal anxiety level, 8-15 a mild anxiety level, 16-25 a moderate anxiety level, and 26-63 a severe anxiety level). For the measurement of stress, we used the 10-item PSS [40], where each item is rated on a 0-4 scale, and the total score range is 0 to 40 (scores of 0-13 indicate mild levels of stress, 14-26 moderate, and 27-40 high). The secondary outcomes were quality of life, life satisfaction, and mindfulness. We used the 16-item Quality of Life Scale (QOLS) [41]; each item is rated on a scale from 1 to 7 and the total score ranges from 16 to 112. Student life satisfaction was measured using the 6-item Brief Multidimensional Students' Life Satisfaction Scale-Peabody Treatment Progress Battery (BMSLSS-PTPB) [42]; each item is rated on a scale from 1 to 5, and item scores are averaged together to give a total score that ranges from 1 to 5. The level of mindfulness was measured by the 24-item FFMQ-SF [43]; each item is rated on a scale from 1 to 5, and the total score range is 24 to 120. The subscales in the FFMQ-SF are nonreactivity to inner experience (5 items), observing (4 items), acting with awareness (5 items), describing (5 items), and nonjudging of inner experience (5 items). We assessed each of the scales for internal consistency within the T1, T2, and T3 data sets, and Cronbach α ranged from .82 to .94: .87, .89, and .86 for PHQ-9; .93, .93, and .94 for BAI; .90, .89, and .90 for PSS; .87, .91, and .92 for QOLS; .82, .85, and .85 for BMSLSS-PTPB; and .86, .87, and .89 for FFMQ-SF. Participants also completed a sociodemographic questionnaire at T1 that inquired about age, gender, birth country, years lived in Canada, first language, relationship status, and ethnic heritage.
All outcomes and other variables were measured by the self-report questionnaires at T1 (baseline) and T2 (8 weeks later). The primary RCT outcomes were depression, anxiety, and perceived stress, while mindfulness was measured as a secondary outcome. It was hypothesized that symptom scores for depression, anxiety, and stress at T2 would be significantly lower in the MVC group compared with the WLC group, and that mindfulness scores at T2 would be significantly higher in the MVC group (compared with WLC). The primary outcomes were measured with the following validated scales: PHQ-9 [40]; BAI [41], PSS [42], and FFMQ-SF [43]. Participants also completed a sociodemographic questionnaire at T1."

6a-i) Online questionnaires: describe if they were validated for online use and apply CHERRIES items to describe how the questionnaires were designed/deployed

If outcomes were obtained through online questionnaires, describe if they were validated for online use and apply CHERRIES items to describe how the questionnaires were designed/deployed [9].

	1	2	3	4	5	
subitem not at all important	◉	○	○	○	○	essential

Clear selection

Does your paper address subitem 6a-i?

Copy and paste relevant sections from manuscript text

The questionnaires have been used online but have not been specifically validated for online use.

6a-ii) Describe whether and how “use” (including intensity of use/dosage) was defined/measured/monitored

Describe whether and how “use” (including intensity of use/dosage) was defined/measured/monitored (logins, logfile analysis, etc.). Use/adoption metrics are important process outcomes that should be reported in any ehealth trial.

	1	2	3	4	5	
subitem not at all important	◉	○	○	○	○	essential

Clear selection

Does your paper address subitem 6a-ii?

Copy and paste relevant sections from manuscript text

We did not measure use precisely and for the purpose of this study at this RCT stage do not see 'use dose' as relevant.

6a-iii) Describe whether, how, and when qualitative feedback from participants was obtained

Describe whether, how, and when qualitative feedback from participants was obtained (e.g., through emails, feedback forms, interviews, focus groups).

	1	2	3	4	5	
subitem not at all important	○	○	○	◉	○	essential

Clear selection

Does your paper address subitem 6a-iii?

Copy and paste relevant sections from manuscript text

Not addressed.

6b) Any changes to trial outcomes after the trial commenced, with reasons

Does your paper address CONSORT subitem 6b? *

Copy and paste relevant sections from the manuscript (include quotes in quotation marks "like this" to indicate direct quotes from your manuscript), or elaborate on this item by providing additional information not in the ms, or briefly explain why the item is not applicable/relevant for your study

No changes were made.

7a) How sample size was determined

NPT: When applicable, details of whether and how the clustering by care provides or centers was addressed

7a-i) Describe whether and how expected attrition was taken into account when calculating the sample size

Describe whether and how expected attrition was taken into account when calculating the sample size.

	1	2	3	4	5	
subitem not at all important	○	○	○	●	○	essential

Clear selection

Does your paper address subitem 7a-i?

Copy and paste relevant sections from manuscript title (include quotes in quotation marks "like this" to indicate direct quotes from your manuscript), or elaborate on this item by providing additional information not in the ms, or briefly explain why the item is not applicable/relevant for your study

"The sample size was calculated for 80% power and 5% type I error to detect of standardized effect size of 0.5 or larger. The required sample size was 63 students per arm. Our recruitment goal was set at 80 participants per arm, assuming an attrition rate of ~ 20%"

7b) When applicable, explanation of any interim analyses and stopping guidelines

Does your paper address CONSORT subitem 7b? *

Copy and paste relevant sections from the manuscript (include quotes in quotation marks "like this" to indicate direct quotes from your manuscript), or elaborate on this item by providing additional information not in the ms, or briefly explain why the item is not applicable/relevant for your study

None

8a) Method used to generate the random allocation sequence

NPT: When applicable, how care providers were allocated to each trial group

Does your paper address CONSORT subitem 8a? *

Copy and paste relevant sections from the manuscript (include quotes in quotation marks "like this" to indicate direct quotes from your manuscript), or elaborate on this item by providing additional information not in the ms, or briefly explain why the item is not applicable/relevant for your study

"Participating students were randomized to the MVC intervention or the wait-list (control) using 1:1 block randomization. The randomized allocation sequence was computer-generated by an off-site research team member and allocations were concealed in sequentially numbered opaque envelopes [28]. The envelopes were only opened after written consent was obtained, ensuring participants and staff were blind prior to the allocation."

8b) Type of randomisation; details of any restriction (such as blocking and block size)

Does your paper address CONSORT subitem 8b? *

Copy and paste relevant sections from the manuscript (include quotes in quotation marks "like this" to indicate direct quotes from your manuscript), or elaborate on this item by providing additional information not in the ms, or briefly explain why the item is not applicable/relevant for your study

"Participating students were randomized to the MVC intervention or the wait-list (control) using 1:1 block randomization. "

9) Mechanism used to implement the random allocation sequence (such as sequentially numbered containers), describing any steps taken to conceal the sequence until interventions were assigned

Does your paper address CONSORT subitem 9? *

Copy and paste relevant sections from the manuscript (include quotes in quotation marks "like this" to indicate direct quotes from your manuscript), or elaborate on this item by providing additional information not in the ms, or briefly explain why the item is not applicable/relevant for your study

The randomized allocation sequence was computer-generated by an off-site research team member and allocations were concealed in sequentially numbered opaque envelopes [28]. The envelopes were only opened after written consent was obtained, ensuring participants and staff were blind prior to the allocation."

10) Who generated the random allocation sequence, who enrolled participants, and who assigned participants to interventions

Does your paper address CONSORT subitem 10? *

Copy and paste relevant sections from the manuscript (include quotes in quotation marks "like this" to indicate direct quotes from your manuscript), or elaborate on this item by providing additional information not in the ms, or briefly explain why the item is not applicable/relevant for your study

"An off-site research team member (biostatistician). Allocations were concealed in sequentially numbered opaque envelopes. Research coordinator selected the next opaque envelope to allocate subjects to comparison groups."

11a) If done, who was blinded after assignment to interventions (for example, participants, care providers, those assessing outcomes) and how

NPT: Whether or not administering co-interventions were blinded to group assignment

11a-i) Specify who was blinded, and who wasn't

Specify who was blinded, and who wasn't. Usually, in web-based trials it is not possible to blind the participants [1, 3] (this should be clearly acknowledged), but it may be possible to blind outcome assessors, those doing data analysis or those administering co-interventions (if any).

	1	2	3	4	5	
subitem not at all important	○	○	◉	○	○	essential

Clear selection

Does your paper address subitem 11a-i? *

Copy and paste relevant sections from the manuscript (include quotes in quotation marks "like this" to indicate direct quotes from your manuscript), or elaborate on this item by providing additional information not in the ms, or briefly explain why the item is not applicable/relevant for your study

'The envelopes were only opened after written consent was obtained, ensuring that participants and the research coordinator (and other staff) were blind prior to allocation."

11a-ii) Discuss e.g., whether participants knew which intervention was the "intervention of interest" and which one was the "comparator"

Informed consent procedures (4a-ii) can create biases and certain expectations - discuss e.g., whether participants knew which intervention was the "intervention of interest" and which one was the "comparator".

	1	2	3	4	5	
subitem not at all important	○	○	○	◉	○	essential

Clear selection

Does your paper address subitem 11a-ii?

Copy and paste relevant sections from the manuscript (include quotes in quotation marks "like this" to indicate direct quotes from your manuscript), or elaborate on this item by providing additional information not in the ms, or briefly explain why the item is not applicable/relevant for your study

Student were aware of being allocated to the MVC intervention or to a wait list control group.

11b) If relevant, description of the similarity of interventions

(this item is usually not relevant for ehealth trials as it refers to similarity of a placebo or sham intervention to a active medication/intervention)

Does your paper address CONSORT subitem 11b? *

Copy and paste relevant sections from the manuscript (include quotes in quotation marks "like this" to indicate direct quotes from your manuscript), or elaborate on this item by providing additional information not in the ms, or briefly explain why the item is not applicable/relevant for your study

Not relevant.

12a) Statistical methods used to compare groups for primary and secondary outcomes

NPT: When applicable, details of whether and how the clustering by care providers or centers was addressed

Does your paper address CONSORT subitem 12a? *

Copy and paste relevant sections from the manuscript (include quotes in quotation marks "like this" to indicate direct quotes from your manuscript), or elaborate on this item by providing additional information not in the ms, or briefly explain why the item is not applicable/relevant for your study

Descriptive statistics for demographic and psychological characteristics were calculated at baseline, and potential between-group differences were assessed using chi-square tests of independence for categorical variables, and t tests for numeric variables. To evaluate the impact of the intervention on study outcomes (ie, depression, anxiety, perceived stress, and mindfulness), generalized estimating equations with an AR (1) covariance structure were employed. Outcome evaluations were performed on both an intention-to-treat (ITT) and per-protocol (PP) basis, with the group × time interaction indicating a between-group change in study outcomes. As the missing data were minimal (<10% overall) and considered to be missing at random, multiple imputations were used to estimate missing observations. For all outcomes, effect sizes are presented for between-group comparisons at follow-up, evaluated by Cohen d and calculated as difference between MVC and WLC means divided by their pooled standard deviations.

12a-i) Imputation techniques to deal with attrition / missing values

Imputation techniques to deal with attrition / missing values: Not all participants will use the intervention/comparator as intended and attrition is typically high in ehealth trials. Specify how participants who did not use the application or dropped out from the trial were treated in the statistical analysis (a complete case analysis is strongly discouraged, and simple imputation techniques such as LOCF may also be problematic [4]).

	1	2	3	4	5	
subitem not at all important	○	○	○	●	○	essential

Clear selection

Does your paper address subitem 12a-i? *

Copy and paste relevant sections from the manuscript (include quotes in quotation marks "like this" to indicate direct quotes from your manuscript), or elaborate on this item by providing additional information not in the ms, or briefly explain why the item is not applicable/relevant for your study

"As the missing data were minimal (<10% overall) and considered to be missing at random, multiple imputations were used to estimate missing observations.

12b) Methods for additional analyses, such as subgroup analyses and adjusted analyses

Does your paper address CONSORT subitem 12b? *

Copy and paste relevant sections from the manuscript (include quotes in quotation marks "like this" to indicate direct quotes from your manuscript), or elaborate on this item by providing additional information not in the ms, or briefly explain why the item is not applicable/relevant for your study

Tables 5 and 6 show additional analyses that focused on discerning which subgroups were affected more or less by the intervention (given varying baseline depression, anxiety, and perceived stress levels). Across 3 levels of depressive symptoms per PHQ-9 categories (ie, mild, moderate, and severe symptom levels), intervention group reductions were greater than those of the control group. An exception was the moderately severe category, where the greater effect size observed was in the controls. We have highlighted the Cohen d effect sizes to facilitate comparisons. The moderately severe category has modest effect size differences.

Table 5. Analysis of Patient Health Questionnaire-9 subgroups: intervention.

Patient Health Questionnaire-9, intervention	Minimal (0-4)	Mild (5-9)	Moderate (10-14)	Moderately severe (15-19)	Severe (20-27)
	N=20	N=19	N=20	N=11	N=6
Mean (T1/T2)	2.20/4.86	6.84/5.41	11.95/8.79	16.54/12.70	23.33/12.33
Median, Cohen d	2.66, 0.64	–1.43, 0.50	–3.16, 0.67	–3.84, 0.55	–11.0, 2.18

Table 6. Analysis of Patient Health Questionnaire-9 subgroups: control.

Patient Health Questionnaire-9, wait-list control	Minimal (0-4)	Mild (5-9)	Moderate (10-14)	Moderately severe (15-19)	Severe (20-27)
	N=17	N=21	N=26	N=10	N=4
Mean (T1/T2)	1.82/2.17	7.19/6.52	11.61/10.53	16.90/12.41	22.50/14.00
Median, Cohen d	0.35, 0.20	–0.67, 0.19	–1.08, 0.29	–4.49, 0.68	–8.5, 1.52

When similar analyses involved female-male comparisons, the effect sizes and mean reductions were greater in females across mild, moderate, moderately severe, and severe categories. Reductions in cell size are noted, particularly for the male data, as the female study sample far exceeded the male sample (Tables 7 and 8).

Table 7. Analysis of Patient Health Questionnaire-9 subgroups: male

Table 7. Analysis of Patient Health Questionnaire-9 subgroups: male.

Patient Health Questionnaire-9, intervention, females	Minimal (0-4)	Mild (5-9)	Moderate (10-14)	Moderately severe (15-19)	Severe (20-27)
	N=14	N=16	N=13	N=9	N=5
Mean (T1/T2)	2.36/3.37	7.00/5.31	11.92/7.82	16.44/12.19	22.60/11.00
Median, Cohen d	1.01, 0.44	–1.69, 0.56	–4.1, 1.24	–4.25, 0.58	–11.6, 3.07

Table 8. Analysis of Patient Health Questionnaire-9 subgroups: female.

Patient Health Questionnaire-9, intervention, males	Minimal (0-4)	Mild (5-9)	Moderate (10-14)	Moderately severe (15-19)	Severe (20-27)
	N=5	N=3	N=7	N=2	N=1
Mean (T1/T2)	2.00/5.00	6.00/5.93	12.00/10.57	17.00/15.00	27.00/19.00
Median, Cohen d	3.00, 0.97	–0.07, 0.33	–1.43, 0.18	–2.00, 0.37	–8.00, N/A

In established BAI categories (ie, low, moderate, and severe symptoms) intervention group reductions were less than control reductions in both low and high symptom categories, while in the moderate symptom category, intervention reductions were greater (Tables 9 and 10). When analyses focused on comparing females with males, the effect sizes and mean reductions were greater in females across moderate and high categories, while the low category results can be characterized as equal. There were reductions in cell size due to the female-male comparisons (Tables 11 and 12).

Table 9. Analysis of Beck Anxiety Inventory categories: intervention.

Beck Anxiety Inventory, intervention	Low (0-21)	Moderate (22-35)	High (≥36)
	N=49	N=16	N=11
Mean (T1/T2)	8.06/8.41	26.00/17.29	41.54/21.34
Median, Cohen d	0.35, 0.05	–8.71, 1.27	–20.21, 1.72

Table 10. Analysis of Beck Anxiety Inventory categories: control.

Beck Anxiety Inventory, wait-list controls	Low (0-21)	Moderate (22-35)	High (≥36)
	N=51	N=19	N=8
Mean (T1/T2)	8.90/11.02	28.05/20.77	43.00/22.62
Median, Cohen d	2.12, 0.22	–7.28, 0.93	–20.38, 2.31

Table 11. Analysis of Beck Anxiety Inventory categories: female.

Beck Anxiety Inventory, intervention, females	Low (0-21)	Moderate (22-35)	High (≥36)
	N=36	N=14	N=7
Mean (T1/T2)	8.42/8.32	26.14/16.54	43.14/21.53
Median, Cohen d	−0.10, 0.01	−9.60, 1.38	−21.61, 1.84

Table 12. Analysis of Beck Anxiety Inventory categories: male.

Beck Anxiety Inventory, intervention, males	Low (0-21)	Moderate (22-35)	High (≥36)
	N=12	N=2	N=4
Mean (T1/T2)	7.50/8.05	25.0/22.5	38.75/21.00
Median, Cohen d	0.55, 0.07	−2.50, 0.16	−17.75, 0.77

In perceived stress, intervention and control comparisons demonstrated, at the moderate and high stress levels, greater reductions in the intervention group with approximately equal reductions observed at the low level (Tables 13 and 14). When analyses focused on female-male comparisons, in the moderate and high levels, females demonstrated more change than males, while in the low group females had a negative intervention effect (their stress increased) while males had no intervention effects (ie, neither positive or negative; Tables 15 and 16).

Table 13. Analysis of Perceived Stress Scale categories: intervention.

Perceived Stress Scale, intervention	Low (0-13)	Moderate (14-26)	High (27-40)
	N=11	N=44	N=21
Mean (T1/T2)	9.55/11.09	20.25/17.93	30.86/23.36
Median, Cohen d	1.54, 0.26	−2.32, 0.40	−7.5, 1.59

Table 14. Analysis of Perceived Stress Scale categories: control.

Perceived Stress Scale, wait-list controls	Low (0-13)	Moderate (14-26)	High (27-40)
	N=14	N=46	N=18
Mean (T1/T2)	9.14/10.64	20.78/20.57	30.72/26.28
Median, Cohen d	1.5, 0.28	−0.21, 0.04	−4.44, 0.88

Table 15. Analysis of Perceived Stress Scale categories: female.

Perceived Stress Scale, intervention, female	Low (0-13)	Moderate (14-26)	High (27-40)
	N=8	N=33	N=16
Mean (T1/T2)	8.87/11.0	19.97/17.55	31.25/23.41
Median, Cohen d	2.13, 0.50	−2.42, 0.42	−7.84, 1.77

Table 16. Analysis of Perceived Stress Scale categories: male.

Perceived Stress Scale, intervention, males	Low (0-13)	Moderate (14-26)	High (27-40)
	N=3	N=11	N=4
Mean (T1/T2)	11.33/11.33	21.09/19.06	30.0/21.25
Median, Cohen d	0.00, 0	−2.03, 0.33	−8.75, 1.33

Altogether, females gained more benefit than males and intervention subjects benefitted more than controls. Gains were most evident in depression and perceived stress reductions. Anxiety results (BAI outcomes) were more variable, likely reflecting the immediate anxiety reduction effects in controls with the suspension of expected exams and assignments.

X26) REB/IRB Approval and Ethical Considerations [recommended as subheading under "Methods"] (not a CONSORT item)

X26-i) Comment on ethics committee approval

	1	2	3	4	5	
subitem not at all important	○	○	○	○	◉	essential

Clear selection

Does your paper address subitem X26-i?

Copy and paste relevant sections from the manuscript (include quotes in quotation marks "like this" to indicate direct quotes from your manuscript), or elaborate on this item by providing additional information not in the ms, or briefly explain why the item is not applicable/relevant for your study

"This study was a two-arm parallel-design RCT comparing the web-based Mindfulness Virtual Community program to a wait-list control group. The Human Participation Research Committee at York University provided research ethics approval for the RCT (Certificate number: e2016-345)."

x26-ii) Outline informed consent procedures

Outline informed consent procedures e.g., if consent was obtained offline or online (how? Checkbox, etc.?), and what information was provided (see 4a-ii). See [6] for some items to be included in informed consent documents.

	1	2	3	4	5	
subitem not at all important	○	○	○	●	○	essential

Clear selection

Does your paper address subitem X26-ii?

Copy and paste relevant sections from the manuscript (include quotes in quotation marks "like this" to indicate direct quotes from your manuscript), or elaborate on this item by providing additional information not in the ms, or briefly explain why the item is not applicable/relevant for your study

Eligible and willing students received detailed in-person information about the study and provided informed written consent. Participants had the option to receive an honorarium of Can $50 (US $39) or 2% in course grade (for professors who gave permission for this option) or three credits (equivalent to 2% course grade) in the Undergraduate Research Participation Pool (URPP) of the Department of Psychology. Each participant also received a resource list that included information about health and social services on campus and in the community (eg, the 24/7 “Good to Talk” helpline for postsecondary students in Ontario). Our protocol included a safety mechanism whereby participants were asked verbally and on the consent form to contact the research staff if they felt distress during the trial period so that “limited counselling with a clinical psychologist could be arranged, if needed.” No such requests arose during the reported study period.

X26-iii) Safety and security procedures

Safety and security procedures, incl. privacy considerations, and any steps taken to reduce the likelihood or detection of harm (e.g., education and training, availability of a hotline)

	1	2	3	4	5	
subitem not at all important	○	○	○	○	◉	essential

Clear selection

Does your paper address subitem X26-iii?

Copy and paste relevant sections from the manuscript (include quotes in quotation marks "like this" to indicate direct quotes from your manuscript), or elaborate on this item by providing additional information not in the ms, or briefly explain why the item is not applicable/relevant for your study

Our protocol included a safety mechanism whereby participants were asked verbally and on the consent form to contact the research staff if they felt distress during the trial period so that “limited counselling with a clinical psychologist could be arranged, if needed.” No such requests arose during the reported study period.

RESULTS

13a) For each group, the numbers of participants who were randomly assigned, received intended treatment, and were analysed for the primary outcome

NPT: The number of care providers or centers performing the intervention in each group and the number of patients treated by each care provider in each center

Does your paper address CONSORT subitem 13a? *

Copy and paste relevant sections from the manuscript (include quotes in quotation marks "like this" to indicate direct quotes from your manuscript), or elaborate on this item by providing additional information not in the ms, or briefly explain why the item is not applicable/relevant for your study

Study participants were 154 undergraduate university students randomized to MVC (n=76) or WLC (n=78) conditions. Of the 154 participants with complete baseline assessments, 7 participants (9.2%) within the MVC and 1 (1.3%) WLC participant dropped out of the study without completing follow-up assessments.

13b) For each group, losses and exclusions after randomisation, together with reasons

Does your paper address CONSORT subitem 13b? (NOTE: Preferably, this is shown in a CONSORT flow diagram) *

Copy and paste relevant sections from the manuscript (include quotes in quotation marks "like this" to indicate direct quotes from your manuscript), or elaborate on this item by providing additional information not in the ms, or briefly explain why the item is not applicable/relevant for your study

Study participants were 154 undergraduate university students randomized to MVC (n=76) or WLC (n=78) conditions. Of the 154 participants with complete baseline assessments, 7 participants (9.2%) within the MVC and 1 (1.3%) WLC participant dropped out of the study without completing follow-up assessments.

13b-i) Attrition diagram

Strongly recommended: An attrition diagram (e.g., proportion of participants still logging in or using the intervention/comparator in each group plotted over time, similar to a survival curve) or other figures or tables demonstrating usage/dose/engagement.

	1	2	3	4	5	
subitem not at all important	○	◉	○	○	○	essential

Clear selection

Does your paper address subitem 13b-i?

Copy and paste relevant sections from the manuscript or cite the figure number if applicable (include quotes in quotation marks "like this" to indicate direct quotes from your manuscript), or elaborate on this item by providing additional information not in the ms, or briefly explain why the item is not applicable/relevant for your study

Attrition data is presented in Table format.

14a) Dates defining the periods of recruitment and follow-up

Does your paper address CONSORT subitem 14a? *

Copy and paste relevant sections from the manuscript (include quotes in quotation marks "like this" to indicate direct quotes from your manuscript), or elaborate on this item by providing additional information not in the ms, or briefly explain why the item is not applicable/relevant for your study

A sample of 480 students (160 students per group) was recruited over 3 semesters (Fall 2017, Winter 2018, and Fall 2018). The 3 samples were not combined due to the campus environment differences related to the 3-month strike in Winter 2018. Here, we report on a sample of n=154 students in a two-arm RCT.

14a-i) Indicate if critical “secular events” fell into the study period

Indicate if critical “secular events” fell into the study period, e.g., significant changes in Internet resources available or “changes in computer hardware or Internet delivery resources”

	1	2	3	4	5	
subitem not at all important	○	○	●	○	○	essential

Clear selection

Does your paper address subitem 14a-i?

Copy and paste relevant sections from the manuscript (include quotes in quotation marks "like this" to indicate direct quotes from your manuscript), or elaborate on this item by providing additional information not in the ms, or briefly explain why the item is not applicable/relevant for your study

The only relevant secular event was the 3 month strike in Winter, 2018.
"A sample of 480 students (160 students per group) was recruited over 3 semesters (Fall 2017, Winter 2018, and Fall 2018). The 3 samples were not combined due to the campus environment differences related to the 3-month strike in Winter 2018. Here, we report on a sample of n=154 students in a two-arm RCT."

14b) Why the trial ended or was stopped (early)

Does your paper address CONSORT subitem 14b? *

Copy and paste relevant sections from the manuscript (include quotes in quotation marks "like this" to indicate direct quotes from your manuscript), or elaborate on this item by providing additional information not in the ms, or briefly explain why the item is not applicable/relevant for your study

NA

15) A table showing baseline demographic and clinical characteristics for each group

NPT: When applicable, a description of care providers (case volume, qualification, expertise, etc.) and centers (volume) in each group

Does your paper address CONSORT subitem 15? *

Copy and paste relevant sections from the manuscript (include quotes in quotation marks "like this" to indicate direct quotes from your manuscript), or elaborate on this item by providing additional information not in the ms, or briefly explain why the item is not applicable/relevant for your study

Table 2. Participants' demographic characteristics at baseline.

Demographic characteristics	All (n=154)	Mindfulness Virtual Community (n=76)	Wait-list control (n=78)	P value
Age (years), mean (SD)a	23.10 (8.09)	22.02 (5.52)	24.18 (9.95)	.10
Gender, n (%)				
Male	35 (22.7)	18 (23.7)	17 (21.8)	.96
Female	117 (76.0)	57 (75.0)	60 (76.9)	
Other	2 (1.3)	1 (1.3)	1 (1.3)	
Country of birth, n (%)				
Canada	91 (59.1)	46 (60.5)	45 (57.7)	.72
Other	63 (40.9)	30 (39.5)	33 (42.3)	
Years in Canada, mean (SD)b	10.73 (7.05)	11.05 (6.21)	10.42 (7.82)	.73
First language, n (%)				
English	103 (66.9)	50 (65.8)	53 (67.9)	.78
Other	51 (33.1)	26 (34.2)	25 (32.1)	
Relationship status, n (%)				
Single, not in a relationship	79 (51.3)	40 (52.6)	39 (50.0)	.70
Single, in a relationship	56 (36.4)	27 (35.5)	29 (37.2)	
Married or common law	13 (8.4)	5 (6.6)	8 (10.3)	
Divorced, separated, widowed, or other	6 (3.9)	4 (5.3)	2 (2.6)	
Ethnicity, n (%)				
White	40 (26.0)	17 (22.4)	23 (29.5)	.69
Black	25 (16.2)	11 (14.5)	14 (17.9)	
South Asian	37 (24.0)	21 (27.6)	16 (20.5)	
Other	35 (22.7)	19 (25.0)	16 (20.5)	
Multiple ethnicities	17 (11.0)	8 (10.5)	9 (11.5)	
Self-rated health, n (%)				
Poor or fair	39 (25.3)	23 (30.3)	16 (20.5)	.38
Good	60 (39.0)	28 (36.8)	32 (41.0)	
Very good or excellent	55 (35.7)	25 (32.9)	30 (38.5)	
Access to private mental health care, n (%)				
Yes	61 (39.6)	31 (40.8)	30 (38.5)	.77
No	93 (60.4)	45 (59.2)	48 (61.5)	
Weekly hours of activities, mean (SD)				
Paid work	7.78 (10.60)	7.76 (9.22)	7.81 (11.84)	.98
Unpaid work (including volunteer work)	[illegible]	[illegible]	[illegible]	

Unpaid work (including volunteer work) 2.87 (5.42) 2.89 (5.81) 2.85 (5.04)
.96
Vigorous physical activitiesc 1.68 (1.87) 1.75 (1.76) 1.62 (1.97) .66
Patient Health Questionnaire score, mean (SD) 9.60 (6.14) 9.53 (5.83) 9.67
(6.50) .88
0-9 77 (50.0) 39 (51.3) 38 (48.7) .74
≥10 77 (50.0) 37 (48.7) 40 (51.3)
Beck Anxiety Inventory score, mean (SD) 16.88 (13.32) 16.68 (13.56)
17.06 (13.17) .86
0-21 (low) 100 (65.0) 49 (64.5) 51 (65.4) .69
22-35 (moderate) 35 (22.7) 16 (21.0) 19 (24.4)
≥36 (high) 19 (12.3) 11 (14.5) 8 (10.2)
Perceived Stress Scale score, mean (SD) 21.30 (7.66) 21.63 (7.72) 20.99
(7.63) .60
0-13 (low) 25 (16.2) 11 (14.5) 14 (17.9) .74
14-26 (moderate) 90 (58.4) 44 (57.9) 46 (59.0)
27-40 (high) 39 (25.3) 21 (27.6) 18 (23.1)
Five-Facet Mindfulness Questionnaire score, mean (SD) 74.15 (12.42) 73.36
(11.22) 74.92 (13.52) .44
aBased on n=152 participants (missing data on n=2).
bBased on n=63 participants born outside of Canada.
cBased on n=151 participants (missing data on n=3).

Table 3 shows the means and standard deviations for study outcomes at baseline and at 8-week follow-up for the MVC and WLC groups. Table 3 further includes effect sizes for the mean difference between MVC and WLC at 8-week follow up. The PHQ-9 and the FFMQ-SF exhibited negligible between-group differences at 8 weeks, while the between-group effect sizes for the BAI, PSS, and FFMQ-SF nonjudgment subscale were in the small range (d=0.20-0.24).

Table 3. Descriptive statistics for depression, anxiety, stress, and mindfulness scores.
Outcomes Intention to treat Per protocol
Mindfulness Virtual Community, mean (SD) Wait-list control, mean (SD)
Cohen d Mindfulness Virtual Community, mean (SD) Wait-list control, mean (SD)
Cohen d
n=76 n=78 n=69 n=77

Patient Health Questionnaire-9
Baseline 9.67 (6.50) 9.53 (5.83) N/Aa 9.80 (6.63) 9.42 (5.78) N/A
8 weeks 7.76 (6.12) 8.05 (6.25) 0.05 7.81 (6.41) 8.05 (6.30) 0.04
[illegible]

Beck Anxiety Inventory						
Baseline	16.68 (13.56)	17.06 (13.17)	N/A	16.91 (13.56)	16.91 (13.19)	N/A
8 weeks	12.15 (10.50)	14.58 (12.29)	0.21	12.29 (10.84)	14.61 (12.37)	0.20
Perceived Stress Scale						
Baseline	21.63 (7.72)	20.99 (7.63)	N/A	21.52 (7.74)	20.99 (7.68)	N/A
8 weeks	18.44 (7.51)	20.11 (7.83)	0.22	18.28 (7.82)	20.12 (7.88)	0.24
Five Facets Mindfulness Questionnaire-Short Form						
Baseline	73.35 (11.22)	74.92 (13.52)	N/A	73.22 (11.10)	74.92 (13.61)	N/A
8 weeks	75.65 (10.08)	75.89 (12.49)	0.02	75.62 (10.58)	75.89 (12.57)	0.02
Five Facets Mindfulness Questionnaire-Short Form, Nonreactivity subscale						
Baseline	14.33 (3.79)	14.22 (4.31)	N/A	14.20 (3.77)	14.20 (4.34)	N/A
8 weeks	14.65 (3.26)	14.69 (3.58)	0.01	14.67 (3.39)	14.68 (3.60)	0.003
Five Facets Mindfulness Questionnaire-Short Form, Observing subscale						
Baseline	13.38 (3.31)	13.65 (3.64)	N/A	13.38 (3.33)	13.69 (3.65)	N/A
8 weeks	13.46 (3.18)	13.90 (3.43)	0.13	13.45 (3.33)	13.90 (3.45)	0.13
Five Facets Mindfulness Questionnaire-Short Form, Acting with Awareness subscale						
Baseline	15.54 (4.12)	15.92 (4.08)	N/A	15.72 (4.10)	15.92 (4.11)	N/A
8 weeks	16.10 (3.87)	16.39 (4.08)	0.07	16.09 (4.05)	16.40 (4.11)	0.08
Five Facets Mindfulness Questionnaire-Short Form, Describing subscale						
Baseline	16.32 (4.16)	16.89 (4.15)	N/A	16.22 (4.30)	16.90 (4.17)	N/A
8 weeks	16.92 (3.51)	17.19 (4.26)	0.07	16.90 (3.68)	17.18 (4.29)	0.07
Five Facets Mindfulness Questionnaire-Short Form, Nonjudgement subscale						
Baseline	13.79 (3.28)	14.24 (3.99)	N/A	13.69 (3.28)	14.22 (4.01)	N/A
8 weeks	14.52 (3.48)	13.72 (3.69)	0.22	14.52 (3.65)	13.73 (3.71)	0.22

aN/A: not applicable.

15-i) Report demographics associated with digital divide issues

In ehealth trials it is particularly important to report demographics associated with digital divide issues, such as age, education, gender, social-economic status, computer/Internet/ehealth literacy of the participants, if known.

	1	2	3	4	5	
subitem not at all important	○	○	○	◉	○	essential

Clear selection

Does your paper address subitem 15-i? *

Copy and paste relevant sections from the manuscript (include quotes in quotation marks "like this" to indicate direct quotes from your manuscript), or elaborate on this item by providing additional information not in the ms, or briefly explain why the item is not applicable/relevant for your study

These subgroups were reported although sample size restraints prevented analyses.

16) For each group, number of participants (denominator) included in each analysis and whether the analysis was by original assigned groups

16-i) Report multiple "denominators" and provide definitions

Report multiple "denominators" and provide definitions: Report N's (and effect sizes) "across a range of study participation [and use] thresholds" [1], e.g., N exposed, N consented, N used more than x times, N used more than y weeks, N participants "used" the intervention/comparator at specific pre-defined time points of interest (in absolute and relative numbers per group). Always clearly define "use" of the intervention.

	1	2	3	4	5	
subitem not at all important	○	○	○	◉	○	essential

Clear selection

Does your paper address subitem 16-i? *

Copy and paste relevant sections from the manuscript (include quotes in quotation marks "like this" to indicate direct quotes from your manuscript), or elaborate on this item by providing additional information not in the ms, or briefly explain why the item is not applicable/relevant for your study

The relevant tables provide 'N" for each group in the trial under both ITT and PP analyses/

16-ii) Primary analysis should be intent-to-treat

Primary analysis should be intent-to-treat, secondary analyses could include comparing only "users", with the appropriate caveats that this is no longer a randomized sample (see 18-i).

	1	2	3	4	5	
subitem not at all important	○	○	○	◉	○	essential

Clear selection

Does your paper address subitem 16-ii?

Copy and paste relevant sections from the manuscript (include quotes in quotation marks "like this" to indicate direct quotes from your manuscript), or elaborate on this item by providing additional information not in the ms, or briefly explain why the item is not applicable/relevant for your study

The paper utilizes both ITT and PP analyses.

17a) For each primary and secondary outcome, results for each group, and the estimated effect size and its precision (such as 95% confidence interval)

Does your paper address CONSORT subitem 17a? *

Copy and paste relevant sections from the manuscript (include quotes in quotation marks "like this" to indicate direct quotes from your manuscript), or elaborate on this item by providing additional information not in the ms, or briefly explain why the item is not applicable/relevant for your study

We provide effect sizes in table 2; 95% CI and SE for table 4 for both (ITT and PP analytic approaches).

17a-i) Presentation of process outcomes such as metrics of use and intensity of use

In addition to primary/secondary (clinical) outcomes, the presentation of process outcomes such as metrics of use and intensity of use (dose, exposure) and their operational definitions is critical. This does not only refer to metrics of attrition (13-b) (often a binary variable), but also to more continuous exposure metrics such as "average session length". These must be accompanied by a technical description how a metric like a "session" is defined (e.g., timeout after idle time) [1] (report under item 6a).

	1	2	3	4	5	
subitem not at all important	○	○	◉	○	○	essential

Clear selection

Does your paper address subitem 17a-i?

Copy and paste relevant sections from the manuscript (include quotes in quotation marks "like this" to indicate direct quotes from your manuscript), or elaborate on this item by providing additional information not in the ms, or briefly explain why the item is not applicable/relevant for your study

Not applicable at this stage of research.

17b) For binary outcomes, presentation of both absolute and relative effect sizes is recommended

Does your paper address CONSORT subitem 17b? *

Copy and paste relevant sections from the manuscript (include quotes in quotation marks "like this" to indicate direct quotes from your manuscript), or elaborate on this item by providing additional information not in the ms, or briefly explain why the item is not applicable/relevant for your study

NA

18) Results of any other analyses performed, including subgroup analyses and adjusted analyses, distinguishing pre-specified from exploratory

Does your paper address CONSORT subitem 18? *

Copy and paste relevant sections from the manuscript (include quotes in quotation marks "like this" to indicate direct quotes from your manuscript), or elaborate on this item by providing additional information not in the ms, or briefly explain why the item is not applicable/relevant for your study

Between and within group effect size calculations are utilized to compare study groups and sub-groups. Table 2 outlines between group effect sizes at follow up for MVC and WLC groups. In addition, tables 5-16 describe sub-group analyse bsed on symptom severity categories for depression anxiety and stress outcomes according to study group and gender.

18-i) Subgroup analysis of comparing only users

A subgroup analysis of comparing only users is not uncommon in ehealth trials, but if done, it must be stressed that this is a self-selected sample and no longer an unbiased sample from a randomized trial (see 16-iii).

	1	2	3	4	5	
subitem not at all important	○	○	○	○	○	essential

Does your paper address subitem 18-i?

Copy and paste relevant sections from the manuscript (include quotes in quotation marks "like this" to indicate direct quotes from your manuscript), or elaborate on this item by providing additional information not in the ms, or briefly explain why the item is not applicable/relevant for your study

Between and within group effect size calculations are utilized to compare study groups and sub-groups. Table 2 outlines between group effect sizes at follow up for MVC and WLC groups. In addition, tables 5-16 describe sub-group analyse bsed on symptom severity categories for depression anxiety and stress outcomes according to study group and gender.

19) All important harms or unintended effects in each group

(for specific guidance see CONSORT for harms)

Does your paper address CONSORT subitem 19? *

Copy and paste relevant sections from the manuscript (include quotes in quotation marks "like this" to indicate direct quotes from your manuscript), or elaborate on this item by providing additional information not in the ms, or briefly explain why the item is not applicable/relevant for your study

There were no observed harms or unintended effects in any participants.

19-i) Include privacy breaches, technical problems

Include privacy breaches, technical problems. This does not only include physical “harm” to participants, but also incidents such as perceived or real privacy breaches [1], technical problems, and other unexpected/unintended incidents. “Unintended effects” also includes unintended positive effects [2].

	1	2	3	4	5	
subitem not at all important	○	○	○	○	○	essential

Does your paper address subitem 19-i?

Copy and paste relevant sections from the manuscript (include quotes in quotation marks "like this" to indicate direct quotes from your manuscript), or elaborate on this item by providing additional information not in the ms, or briefly explain why the item is not applicable/relevant for your study

No privacy breaches or technical problems.

19-ii) Include qualitative feedback from participants or observations from staff/researchers

Include qualitative feedback from participants or observations from staff/researchers, if available, on strengths and shortcomings of the application, especially if they point to unintended/unexpected effects or uses. This includes (if available) reasons for why people did or did not use the application as intended by the developers.

	1	2	3	4	5	
subitem not at all important	○	○	●	○	○	essential

Clear selection

Does your paper address subitem 19-ii?

Copy and paste relevant sections from the manuscript (include quotes in quotation marks "like this" to indicate direct quotes from your manuscript), or elaborate on this item by providing additional information not in the ms, or briefly explain why the item is not applicable/relevant for your study

NA in this study.

DISCUSSION

22) Interpretation consistent with results, balancing benefits and harms, and considering other relevant evidence

NPT: In addition, take into account the choice of the comparator, lack of or partial blinding, and unequal expertise of care providers or centers in each group

22-i) Restate study questions and summarize the answers suggested by the data, starting with primary outcomes and process outcomes (use)

Restate study questions and summarize the answers suggested by the data, starting with primary outcomes and process outcomes (use).

	1	2	3	4	5	
subitem not at all important	○	◉	○	○	○	essential

Clear selection

Does your paper address subitem 22-i? *

Copy and paste relevant sections from the manuscript (include quotes in quotation marks "like this" to indicate direct quotes from your manuscript), or elaborate on this item by providing additional information not in the ms, or briefly explain why the item is not applicable/relevant for your study

This study was undertaken at a labor strike–affected university, following identical procedures described in two prior studies at the same site, conducted when no crises existed [4,5]. Accordingly, participant responses to the previous RCTs are usefully compared to responses in this study (referred to as “Study 3” below).
In the previously reported RCTs [4,5], significant between-group differences were observed in depression (PHQ-9), anxiety (BAI), quality of life (QOLS), and mindfulness (FFMQ-SF). In the first RCT, significant between-group differences were additionally found in perceived stress (PSS). In this study, a significant between-group difference was only found in the PSS (P=.03), while a marginal (P=.06) difference was observed in the Nonjudgment subscale of FFMQ-SF.
Despite the limited between-group differences in this disrupted-campus study, significant group × time effects suggested that the MVC intervention had a positive impact on students. However, while in Study 1 and 2 the WLC depression scores (PHQ-9) [4,5] increased over time (Time 2>Time 1; perhaps due to anticipating increasingly demanding academic tasks), in Study 3, the WLC subjects had substantially reduced depression scores (Time 1<Time 2, by –1.4 in mean raw score). As the intervention within-group reductions were nearly equivalent across studies 1, 2, and 3, the significant between-group differences absent in Study 3 appeared related to the self-reported depression reductions (between Time 1 and Time 2) in WLC subjects.
Cross-study differences were also observable in the BAI indices, where in Study 1 and 2, WLC scores increased over time (Time 2>Time 1), whereas in Study 3 the WLC scores decreased over time (Time 2<Time 1) by a mean (raw) scale score of –2.38. Once again, the within-group reductions were nearly equivalent across studies 1, 2, and 3 (–4.2, –4.8, and –4.5, respectively) indicating that reductions in the between-group differences in Study 3 were related to anxiety reductions in the WLC group.
Other interesting cross-study differences were observable in PSS indices where significant between-group differences were found in Study 1 and Study 3, but not in Study 2. In Study 1 and 3, the WLC group scores were nearly equivalent (Time 1 and Time 2) as were the reductions achieved in the intervention group (Study 1: –3.10, Study 2: –3.19, in raw scores).
One interpretation of such study comparisons is that students experienced the crisis as reducing academic pressure, with the result of them experiencing less distress (represented in lower mean WLC group depression and anxiety scores).
[illegible]

While the lowered distress was likely temporary (outcomes were assessed 8 weeks after baseline, and prior to resumed course activity), the pattern might have also affected motivations regarding participation in the intervention. If so, this would have contributed to the lack of significant between-group differences in depression and anxiety in this study.

22-ii) Highlight unanswered new questions, suggest future research

Highlight unanswered new questions, suggest future research.

	1	2	3	4	5	
subitem not at all important	○	◉	○	○	○	essential

Clear selection

Does your paper address subitem 22-ii?

Copy and paste relevant sections from the manuscript (include quotes in quotation marks "like this" to indicate direct quotes from your manuscript), or elaborate on this item by providing additional information not in the ms, or briefly explain why the item is not applicable/relevant for your study

NA in this study

20) Trial limitations, addressing sources of potential bias, imprecision, and, if relevant, multiplicity of analyses

20-i) Typical limitations in ehealth trials

Typical limitations in ehealth trials: Participants in ehealth trials are rarely blinded. Ehealth trials often look at a multiplicity of outcomes, increasing risk for a Type I error. Discuss biases due to non-use of the intervention/usability issues, biases through informed consent procedures, unexpected events.

	1	2	3	4	5	
subitem not at all important	○	○	○	◉	○	essential

Clear selection

Does your paper address subitem 20-i? *

Copy and paste relevant sections from the manuscript (include quotes in quotation marks "like this" to indicate direct quotes from your manuscript), or elaborate on this item by providing additional information not in the ms, or briefly explain why the item is not applicable/relevant for your study

Several study limitations must be taken into account. This was not a single- or double-blinded trial. The study duration of 8 weeks did not allow us to test for longer-term effects (eg, 6 or 12 months), which would have resulted in a more fully assessed crisis effect. A further limitation is the high female preponderance in the control and intervention groups, as more precise future research projects would address gender differences through stratification with larger participant samples from multiple research sites (involving multiple universities and colleges). Missing data was also a limitation, somewhat mitigated by the use of a multiple imputation method. Lastly and importantly, given the marginal between-group differences found in this study in mindfulness self-report (FFMQ-SF), there was no measure of the participants' mindfulness practice external to platform participation.

21) Generalisability (external validity, applicability) of the trial findings

NPT: External validity of the trial findings according to the intervention, comparators, patients, and care providers or centers involved in the trial

21-i) Generalizability to other populations

Generalizability to other populations: In particular, discuss generalizability to a general Internet population, outside of a RCT setting, and general patient population, including applicability of the study results for other organizations

	1	2	3	4	5	
subitem not at all important	○	○	○	◉	○	essential

Clear selection

Does your paper address subitem 21-i?

Copy and paste relevant sections from the manuscript (include quotes in quotation marks "like this" to indicate direct quotes from your manuscript), or elaborate on this item by providing additional information not in the ms, or briefly explain why the item is not applicable/relevant for your study

Several study limitations must be taken into account. This was not a single- or double-blinded trial. The study duration of 8 weeks did not allow us to test for longer-term effects (eg, 6 or 12 months), which would have resulted in a more fully assessed crisis effect. A further limitation is the high female preponderance in the control and intervention groups, as more precise future research projects would address gender differences through stratification with larger participant samples from multiple research sites (involving multiple universities and colleges). Missing data was also a limitation, somewhat mitigated by the use of a multiple imputation method. Lastly and importantly, given the marginal between-group differences found in this study in mindfulness self-report (FFMQ-SF), there was no measure of the participants’ mindfulness practice external to platform participation.

21-ii) Discuss if there were elements in the RCT that would be different in a routine application setting

Discuss if there were elements in the RCT that would be different in a routine application setting (e.g., prompts/reminders, more human involvement, training sessions or other co-interventions) and what impact the omission of these elements could have on use, adoption, or outcomes if the intervention is applied outside of a RCT setting.

	1	2	3	4	5	
subitem not at all important	○	◉	○	○	○	essential

Clear selection

Does your paper address subitem 21-ii?

Copy and paste relevant sections from the manuscript (include quotes in quotation marks "like this" to indicate direct quotes from your manuscript), or elaborate on this item by providing additional information not in the ms, or briefly explain why the item is not applicable/relevant for your study

NA in this study.

OTHER INFORMATION

23) Registration number and name of trial registry

Does your paper address CONSORT subitem 23? *

Copy and paste relevant sections from the manuscript (include quotes in quotation marks "like this" to indicate direct quotes from your manuscript), or elaborate on this item by providing additional information not in the ms, or briefly explain why the item is not applicable/relevant for your study

Trial Registration: International Standard Randomized Controlled Trial Number ISRCTN92827275;
https://www.isrctn.com/ISRCTN92827275

24) Where the full trial protocol can be accessed, if available

Does your paper address CONSORT subitem 24? *

Cite a Multimedia Appendix, other reference, or copy and paste relevant sections from the manuscript (include quotes in quotation marks "like this" to indicate direct quotes from your manuscript), or elaborate on this item by providing additional information not in the ms, or briefly explain why the item is not applicable/relevant for your study

NA

25) Sources of funding and other support (such as supply of drugs), role of funders

Does your paper address CONSORT subitem 25? *

Copy and paste relevant sections from the manuscript (include quotes in quotation marks "like this" to indicate direct quotes from your manuscript), or elaborate on this item by providing additional information not in the ms, or briefly explain why the item is not applicable/relevant for your study

No other funders except for the Canadian Institutes for Health Research

X27) Conflicts of Interest (not a CONSORT item)

X27-i) State the relation of the study team towards the system being evaluated

In addition to the usual declaration of interests (financial or otherwise), also state the relation of the study team towards the system being evaluated, i.e., state if the authors/evaluators are distinct from or identical with the developers/sponsors of the intervention.

	1	2	3	4	5	
subitem not at all important	○	○	○	●	○	essential

Clear selection

Does your paper address subitem X27-i?

Copy and paste relevant sections from the manuscript (include quotes in quotation marks "like this" to indicate direct quotes from your manuscript), or elaborate on this item by providing additional information not in the ms, or briefly explain why the item is not applicable/relevant for your study

CE, FA and PR designed the study and study-specific questionnaire items, received and administered funds and contributed equally on most components. PR led the module development, providing written content and voice. MP undertook the statistical analyses CE verified analysis, and PR provided the first draft. All authors provided critical feedback relevant to the revisions undertaken.

About the CONSORT EHEALTH checklist

As a result of using this checklist, did you make changes in your manuscript? *

- ( ) yes, major changes
- ( ) yes, minor changes
- (•) no

What were the most important changes you made as a result of using this checklist?

Your answer

How much time did you spend on going through the checklist INCLUDING making changes in your manuscript *

2.5 hours

As a result of using this checklist, do you think your manuscript has improved? *

- ○ yes
- ◉ no
- ○ Other:

Would you like to become involved in the CONSORT EHEALTH group?

This would involve for example becoming involved in participating in a workshop and writing an "Explanation and Elaboration" document

- ○ yes
- ◉ no
- ○ Other:

Clear selection

Any other comments or questions on CONSORT EHEALTH

There is unnecessary redundancy.

STOP - Save this form as PDF before you click submit

To generate a record that you filled in this form, we recommend to generate a PDF of this page (on a Mac, simply select "print" and then select "print as PDF") before you submit it.

When you submit your (revised) paper to JMIR, please upload the PDF as supplementary file.

Don't worry if some text in the textboxes is cut off, as we still have the complete information in our database. Thank you!

Final step: Click submit !

Click submit so we have your answers in our database!

Submit

Never submit passwords through Google Forms.

This content is neither created nor endorsed by Google. Report Abuse - Terms of Service - Privacy Policy

Google Forms
